# Amplified Spontaneous Emission Enhancement in FAPbI_3_ Nanocrystal Films via PMMA and Mechanical Tunability on Flexible PET

**DOI:** 10.1021/acsami.6c05503

**Published:** 2026-05-21

**Authors:** Ja-Hon Lin, Yu-Ming Li, Kuan-Yu Liao, Chun-Chung Chen, Hsin-Yi Wei, Kai-Wei Lin, Chi-Ching Kuo, Bi-Hsuan Lin, Youhei Chitose, Chihaya Adachi

**Affiliations:** † Department of Electro-Optical Engineering, Advanced Nanophotonics Technology Laboratory, 34877 National Taipei University of Technology, Taipei 106, Taiwan; ‡ Institute of Organic and Polymeric Materials, National Taipei University of Technology, Taipei 106, Taiwan; § 57815 TPS 23A X-ray Nanoprobe Beamline, National Synchrotron Radiation Research Center, Hsinchu 30076, Taiwan; ∥ Center for Organic Photonics and Electronics Research (OPERA), 12923 Kyushu University, 744 Motooka, Nishi, Fukuoka 819-0395, Japan; ⊥ International Institute for Carbon Neutral Energy Research (I2CNER), Kyushu University, 744 Motooka, Nishi, Fukuoka 819-0395, Japan; # Department of Applied Chemistry, Graduate School of Engineering, Center for Molecular Systems(CMS), Kyushu University, Fukuoka 819-0395, Japan; ¶ Organic Photonics and Electronics International Laboratory (OPHELIA), CNRS-Kyushu University, 744 Motooka, Nishi, Fukuoka 819-0395, Japan

**Keywords:** FAPbI_3_ nanocrystals, amplified spontaneous emission, flexible PET substrate, PMMA matrix, mechanical tunability, gain guiding, two-photon absorption

## Abstract

Lead halide perovskites (LHPs), such as FAPbI_3_ nanocrystals (NCs), exhibit suitable bandgap energy and superior thermal and chemical stability, making them ideal for photovoltaic and near-infrared (NIR) laser applications. However, recent studies on NIR amplified spontaneous emission (ASE) from FAPbI_3_ NCs under nanosecond pulsed laser excitation still face the challenge of high lasing thresholds. In this work, FAPbI_3_ NCs were synthesized via the hot-injection method and deposited on a PMMA passivation layer (S-II). Owing to the passivation effect, S-II exhibits enhanced photoluminescence (PL) intensity, a longer carrier lifetime, and higher two-photon absorption (2PA) performance compared to those directly deposited on the glass substrate (S-I). Besides, the improved thermal stability of S-II has been demonstrated based on temperature-dependent ASE measurements, showing a higher characteristic temperature (*T*_C_ ∼176 K). Under both backward- and sideward-scattering configurations, S-II exhibits a lower ASE threshold, higher optical gain, and a higher degree of polarization (DOP), which is attributable to the gain-guiding effect. The flexible ASE of an FAPbI_3_ NC film was produced using PET as the substrate, exhibiting a redshift in the emission peak and an increase in intensity under mechanical-stress-induced bending. The high-performance NIR ASE produced from FAPbI_3_ NCs using PMMA as a passivation layer, combined with mechanical flexibility, demonstrates high potential as a promising gain medium for NIR laser applications.

## Graphical abstract

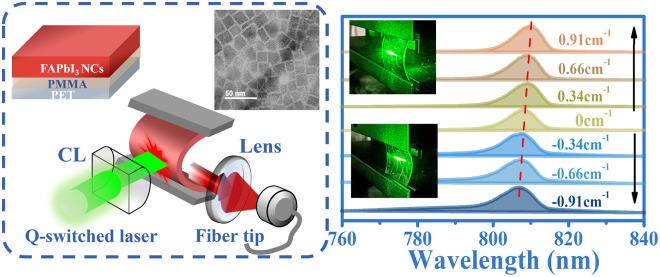

## 1 Introduction

Recently, lead halide perovskites (LHPs) with the chemical formula APbX_3_ [A-site cation: MA^+^(methylammonium, CH_3_NH_3_^+^), FA^+^ (formamidinium, CH­(NH_2_)_2_^+^), or Cs^+^(cesium); X-site anion = I^–^, Br^–^, Cl^–^)] have attracted significant attention as promising materials for a wide range of optoelectronic device applications. The atomic crystal structure of LHPs is constructed by [PbX_6_] octahedra with Pb^2+^ at the center and six X^–^ ions at the vertices. The corner-sharing octahedra form a three-dimensional (3D) network, with the A-site ion occupying the central void to create the 3D perovskite structure. A key benefit of this class of materials for optoelectronic applications is the extensive compositional flexibility at the A- and X-sites, allowing for the fine-tuning of both optical and physical properties. For photovoltaic applications, iodide (I^–^) is commonly employed as the X-site anion due to its relatively narrow bandgap, which facilitates extensive absorption throughout the visible spectral region. Additionally, compared to counterparts such as MAPbI_3_ and CsPbI_3_, FAPbI_3_ exhibits a narrower bandgap of approximately 1.48 eV,^1,2^ which is closer to the Shockley–Queisser limit.^3^ With its long carrier lifetime^4^ and diffusion length,^5^ remarkable thermal stability,^6^ and high absorption coefficient,^5^ FAPbI_3_ demonstrates excellent potential for photovoltaic applications. In addition, FAPbI_3_ thin films have recently received considerable attention as a superior gain medium for near-infrared (NIR) lasers. Under femtosecond (fs) pulsed laser excitation, Yuan et al.^7^ demonstrated low-threshold (∼1.6 μJ/cm^2^) NIR amplified spontaneous emission (ASE) around 812 nm in 2017. Under nanosecond (ns) pulsed laser excitation, Yang et al.^8^ reported NIR ASE (∼820 nm) from FAPbI_3_ thin films synthesized using both polar and nonpolar solvents in 2025. Nevertheless, FAPbI_3_ thin films tend to transition from the optically active α-phase to the nonactive δ-phase at room temperature (RT),^9^ which limits their practical applications. In contrast, other morphologies of FAPbI_3_, such as nanocrystals (NCs), exhibit greater structural stability at RT. Moreover, they exhibit higher photoluminescence quantum yield (PLQY)^10^ and longer carrier lifetimes^11^ compared to their thin-film counterparts, along with a tunable bandgap achieved through size control. Owing to the aforementioned advantages, the ASE characteristics of FAPbI_3_ NCs have also attracted growing attention. Using a dipping method for NC film fabrication and excitation by an fs pulsed laser, Protesescu et al.^12^ reported a low ASE threshold of approximately 7.5 μJ/cm^2^ in 2017. To demonstrate the feasibility of NIR ASE generation under longer ns pulse excitation, an FAPbI_3_ NC film was fabricated using an annealing method in the following year.^13^ However, the observed ASE threshold increased significantly to approximately 140 μJ/cm^2^.

Despite their numerous advantages, hybrid organic–inorganic perovskite NCs still suffer from degradation caused by moisture, oxygen, and prolonged optical excitation.^14–16^ Due to their large surface-to-volume ratio, they also exhibit a high density of surface defect states. To address these issues, various strategies have been proposed, including ligand engineering,^17,18^ post-treatment techniques,^19^ defect repair,^20–22^ and encapsulation methods using different materials such as silicon dioxide (SiO_2_),^23^ metal–organic frameworks (MOFs),^24^ and silk fibroin.^25^ Compared to other materials, cost-effective and easily processable poly­(methyl methacrylate) (PMMA) has been used to reduce the ASE threshold of perovskites and enhance their overall optical performance. For example, covering MAPbI_3_,^26^ FAPbI_3_,^7^ and quasi-2D perovskite thin films^27^ with a PMMA layer has been shown to significantly reduce the ASE threshold. Leveraging the waveguide effect, research groups have incorporated MAPbI_3_ thin films^28^ or CsPbBr_3_ quantum dots (QDs)^29^ within a double-layer PMMA structure, leading to a significant enhancement in ASE performance.

Achieving low-threshold ASE under long-pulse excitation represents a critical step toward continuous-wave (CW) lasing. Although previous studies have reported ASE from FAPbI_3_ NCs using ns Q-switched pulsed lasers as the excitation source, the excitation threshold remains relatively high, around 140 μJ/cm^2^.^13^ Therefore, further reduction of the ASE threshold under ns pulse excitation is necessary. To this end, FAPbI_3_ NCs were synthesized via a feasible hot-injection method and spin-coated onto a PMMA-assisted glass substrate, which served as a passivation layer. To systematically evaluate the effect of the PMMA layer, photoluminescence (PL) measurements under one- and two-photon excitation, along with PL mapping, were performed. In addition, the characteristic temperature was derived from temperature-dependent ASE measurements under a backward-scattering configuration. The degree of polarization (DOP) and gain coefficient were systematically evaluated for FAPbI_3_ NC films with and without the PMMA layer. Finally, a flexible PET substrate was adopted to enable bendable ASE and to systematically investigate the tunability of the lasing characteristics under mechanical deformation.

## 2 Results and Discussion

### 2.1 Properties of FAPbI_3_ NC Films

The transmission electron microscopy (TEM) image in Figure 1a (scale bar: 50 nm) reveals that the synthesized FAPbI_3_ NCs exhibit a well-defined cubic morphology. Based on Gaussian fitting of the corresponding size distribution, as shown in Figure 1b, the average edge length is determined to be 14.4 ± 2.4 nm. A high-resolution transmission electron microscopy (HRTEM) image in Figure 1c clearly reveals lattice fringes, with an observed interplanar spacing of 0.31 nm associated with the (200) reflection plane.^12^ Here, sample S-I was prepared by directly spin-coating FAPbI_3_ NCs onto a glass substrate, while sample S-II involved a predeposited PMMA layer on the glass substrate, followed by the spin-coating of FAPbI_3_ NCs. The structures of the two samples are shown in Figure 1d. The thicknesses of the FAPbI_3_ NC films deposited on the top layers of samples S-I and S-II were measured using a DEKTAKXT surface profiler (Bruker Inc.), as shown in Figure S1 (Supporting Information). Based on measurements taken at five different locations, the average film thicknesses were determined to be approximately 544.6 and 541.6 nm for S-I and S-II, respectively. To assess the surface morphology and uniformity of the films, representative 2D and 3D atomic force microscopy (AFM) images were obtained using a Dimension Icon AFM (Bruker Inc.) under identical scanning conditions to allow direct comparison between samples. The presented images correspond to representative scanned regions of the films. As shown in Figure 1e and f, sample S-II exhibits a smoother and more uniform surface than sample S-I. Based on the data processed using NanoScope Analysis 2.0, the root-mean-square (RMS) roughness values of samples S-I and S-II were determined to be 15.8 and 10.2 nm, respectively. Additionally, the scanning electron microscopy (SEM) images in Figure 1g and h indicate that sample S-I exhibits prominent surface cracks, whereas sample S-II shows a denser and more continuous grain structure. The AFM and SEM results suggest that the incorporation of PMMA improves the structural quality of the deposited FAPbI_3_ NC film, leading to a smoother surface morphology.

**Figure 1:**
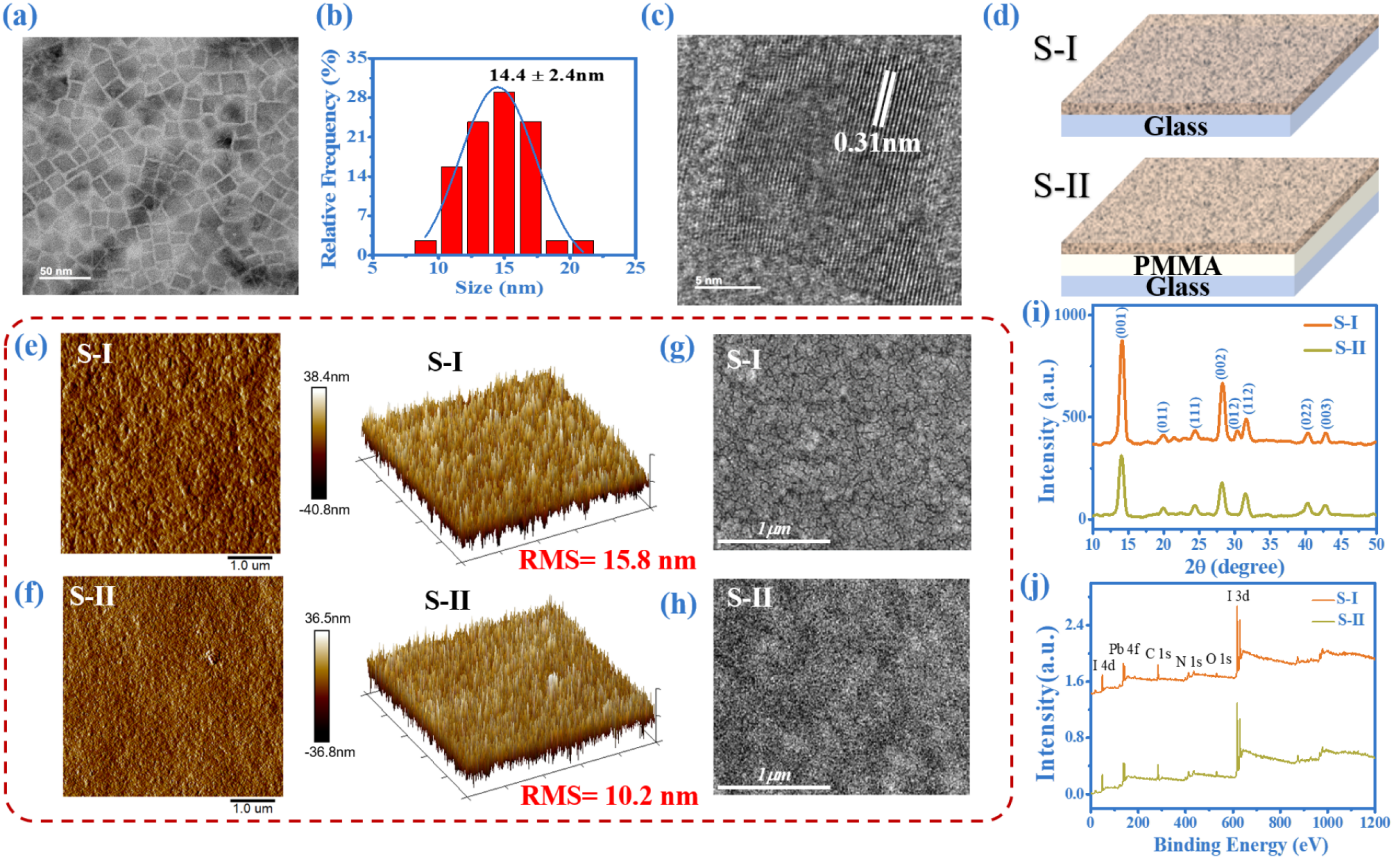
Characteristics of FAPbI_3_ NCs and NC films. (a) TEM image (scale bar: 50 nm) and (b) corresponding size distribution, (c) HRTEM image (scale bar: 5 nm), (d) schematic illustration of the structures of S-I and S-II, 2D and 3D AFM images of (e) S-I and (f) S-II, SEM images of (g) S-I and (h) S-II, (i) XRD patterns (S-I: burnt orange and S-II: olive green); and (j) XPS spectra (S-I: burnt orange and S-II: olive green).

The X-ray diffraction (XRD) patterns shown in Figure 1i confirm that both samples correspond to the cubic α-phase of FAPbI_3_.^30–32^ The X-ray photoelectron spectroscopy (XPS) shown in Figure 1j indicates no significant difference in chemical composition between the two films, with clear characteristic peaks of Pb, C, and I observed. Fine XPS spectra of each element are shown in Figure S2 (Supporting Information), where the peaks at approximately 618.2 and 629.7 eV (I 3d), 284 eV (C 1s), and 142.1 and 137.3 eV (Pb 4f) are in good agreement with literature reports.^33^ In addition, the presence of the O 1s peak indicates partial surface oxidation of the films after exposure to air. To verify the chemical composition of C, N, Pb, and I and their distribution in samples S-I and S-II, energy-dispersive spectroscopy (EDS) images and element mapping are shown in Figure S3­(Supporting Information). The results indicate that the atomic ratio of Pb to I is close to the stoichiometric value of FAPbI_3_ NCs, approximately 1:3.

In Figure 2a the one-photon absorption photoluminescence (1PA PL) spectra, excited by a CW He–Cd laser, show that both samples exhibit emission peaks centered at 1.60 eV (774.8 nm). The peak intensity of sample S-II is significantly higher than that of S-I, which reflects the higher emission efficiency enabled by the underlying PMMA layer. Both samples exhibit a pronounced drop in their absorption spectra below 1.63 eV (760 nm), consistent with their optical band edges obtained from the Tauc plot in Figure S4 (Supporting Information). To further confirm the uniformity of the two FAPbI_3_ NC films, PL mapping and the corresponding histograms demonstrate that S-II exhibits a much narrower distribution of peak wavelength and line width in Figures S5 and S6 (Supporting Information) than the corresponding film on a glass substrate. Time-resolved PL (TRPL) measurements were performed to obtain two-dimensional streak camera images and the corresponding photon decay traces of the FAPbI_3_ NC films, as displayed in Figure 2b and c. Fitting the data with a triexponential model^34,35^ yielded the decay time constants (τ_*i* = 1,2,3_) and their corresponding intensity weightings (*A*_*i* = 1,2,3_), as summarized in Table 1. Here, the decay times were calculated by averaging multiple independent measurements shown in Figures S7 and S8 (Supporting Information), and the corresponding standard deviations are provided. The shorter decay time constants (τ_1_ and τ_2_) correspond to nonradiative processes such as Auger recombination and trap-assisted recombination, while the longer decay time constant τ_3_ is attributed to radiative recombination.^35^ Additionally, the average photon lifetime (τ_*ave*_) was calculated using the following equation:

1 $$\tau_{\mathrm{ave}}=\frac{\overset{m}{\underset{i=1}{\sum }}A_{i}\tau_{i}^{2}}{\overset{m}{\underset{i=1}{\sum }}A_{i}\tau_{i}}=\frac{A_{1}\tau_{1}^{2}+A_{2}\tau_{2}^{2}+A_{3}\tau_{3}^{2}}{A_{1}\tau_{1}+A_{2}\tau_{2}+A_{3}\tau_{3}},,\left(m=3\right)$$

yielding approximately 54.5 ± 4.2 ns for S-I and 62.7 ± 6.3 ns for S-II, which are compatible to the previous results.^36,37^ The longer lifetime observed in S-II is attributed to improved structural quality and a lower density of trap states, which is consistent with a reduced rate of trap-assisted recombination.

**Figure 2:**
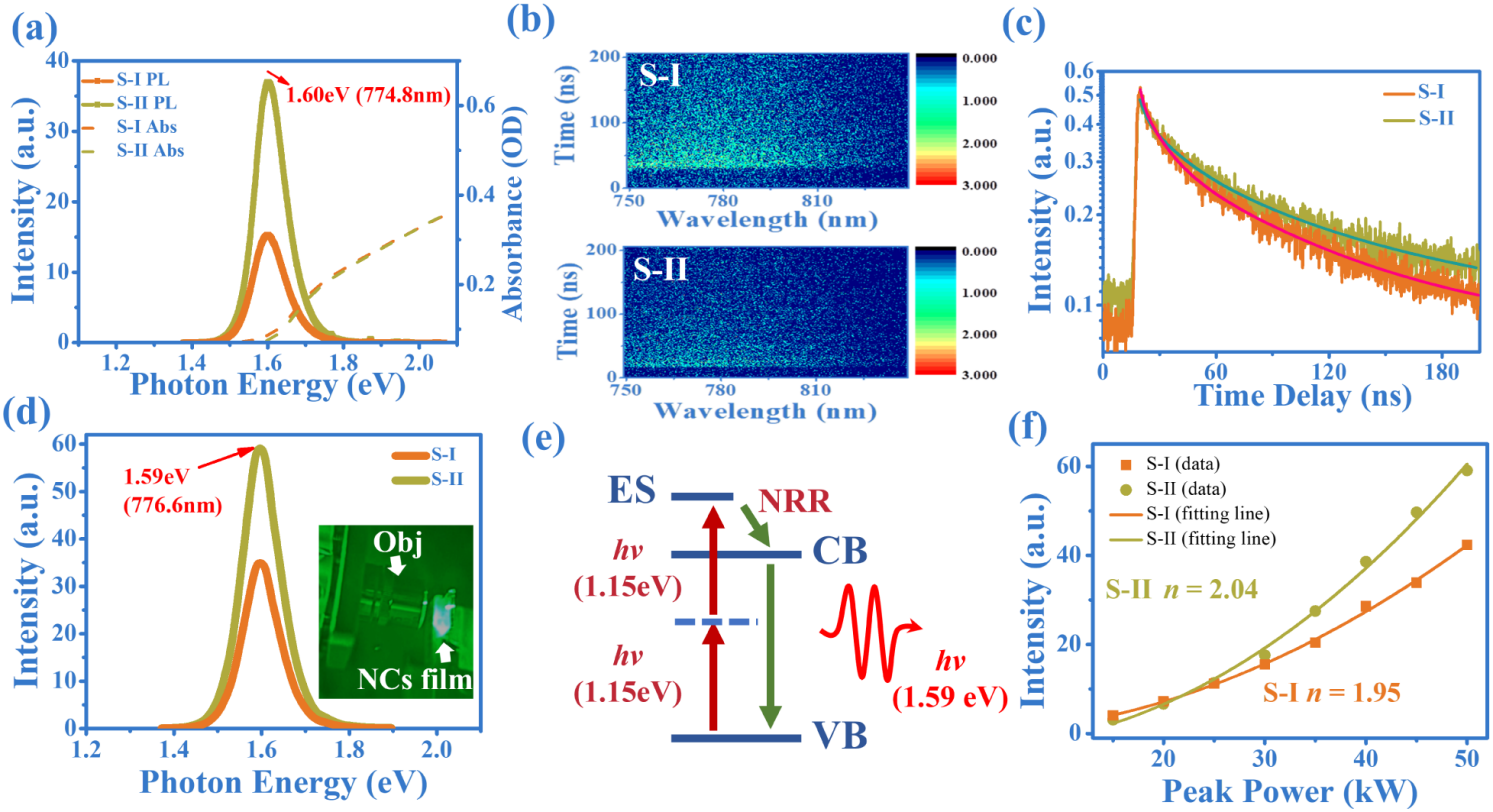
Optical properties of the FAPbI_3_ NC films. (a) PL spectra (S-I: burnt orange solid curve; S-II: olive green solid curve) and absorption spectra (S-I: burnt orange dashed curve; S-II: olive green dashed curve), (b) 2D streak camera images (S-I: top; S-II: bottom), (c) photon decay traces (S-I: burnt orange; S-II: olive green), (d) 2PA PL spectra (S-I: burnt orange; S-II: olive green), with the inset showing a photograph of the sample under fs pulsed laser excitation, captured through an IR viewer, and (e) schematic illustration of the 2PA PL process, and (f) 2PA PL peak intensity as a function of excitation peak power (S-I: burnt orange; S-II: olive green).

**Table 1:** Comparison of TRPL Parameters for FAPbI_3_ NC Film (S-I) and FAPbI_3_ NC/PMMA Film (S-II) from This Work and Previous Reports

sample	τ_1_ (ns)	*A*_1_ (%)	τ_2_ (ns)	*A*_2_ (%)	τ_3_ (ns)	*A*_3_ (%)	τ_ave_ (ns)
FAPbI_3_ PSCs^36^	36.0	94.8	140.1	5.2	-	-	54.2
FAPbI_3_ film^37^	17.0	67.4	64.7	32.6	-	-	47.6
S-I (our work)	1.6 ± 0.3	54.3	12.6 ± 0.9	35.5	82.3 ± 5.1	10.2	54.5 ± 4.2
S-II (our work)	3.4 ± 0.7	59.2	18.7 ± 1.2	27.3	90.8 ± 7.5	13.5	62.7 ± 6.3

Two-photon absorption (2PA) is one of the most important third-order nonlinear optical phenomena and has attracted considerable attention due to its practical applications in areas such as optical limiting,^38^ microfabrication^39,40^ and biomedical imaging.^41^ Consequently, scientists are highly motivated to identify novel materials that exhibit strong 2PA responses. Nevertheless, 2PA-induced PL in FAPbI_3_ NC films has been rarely investigated. Here, the 2PA PL spectra, under excitation by a 1070 nm fs ytterbium-doped fiber laser, are shown in Figure 2d. Similar to the result from 1PA PL, sample S-II exhibits a significantly higher peak intensity than S-I under the same excitation peak power (50 kW) owing to surface passivation by PMMA to reduce the surface trap density. The emission photon energy of both samples, measured at 1.596 eV (776.6 nm), is slightly lower than that obtained from 1PA due to the reabsorption effect.^34^ The inset of Figure 2d shows a photograph of the fs-pulse-excited FAPbI_3_ NC film, captured through the viewing window of an infrared (IR) viewer. The corresponding mechanism of 2PA PL is illustrated by the energy-level diagram in Figure 2e. Carriers are pumped from the valence band (VB) to a high-lying excited state (ES) via the simultaneous absorption of two photons with photon energy of approximately 1.15 eV. This is followed by nonradiative relaxation (NRR) to the bottom of the conduction band (CB), and subsequent photon emission with an energy of approximately 1.59 eV through radiative recombination. The evolution of the PL spectra with increasing pump peak power (*I*_pk_) for the two samples is shown in Figure S9 (Supporting Information). In Figure 2f, the 2PA PL peak intensity (*I*_2PA_) as a function of peak power for the two samples (S-I: burnt-orange squares; S-II: olive-green circles) is well fitted by the power-law expression $I_{\mathrm{2PA}}=\alpha I_{\mathrm{pk}}^{n}$ ,^34,42^ yielding the corresponding fitted curves (S-I: burnt-orange solid line; S-II: olive-green solid line). Since the fitted exponent values *n* for both samples are very close to 2 (1.95 for S-I and 2.04 for S-II), the PL intensity clearly exhibits a quadratic dependence on the excitation power. This quadratic behavior indicates that the emission is predominantly governed by the 2PA mechanism. To further verify the 2PA behavior, the optical limiting properties of the two samples were measured, as shown in Figure S10 (Supporting Information). The results indicate that sample S-II exhibits a stronger optical limiting response than sample S-I, consistent with an enhanced nonlinear absorption effect.

### 2.2 Temperature-Dependent ASE Measurements

As shown in Figure 3a, temperature-dependent ASE measurements were performed by exciting the sample with a 532 nm Q-switched pulsed laser in a backward-scattering configuration. The temperature of the FAPbI_3_ NC film, ranging from 80 to 300 K, was precisely controlled using a cryogenic system (ST-500UC, Janis Inc.). Figure 3b and c shows the ASE peak intensity as a function of pump fluence at RT (300 K). At low pump fluence, the emission spectrum is dominated by broad spontaneous emission (SPE). Upon exceeding the threshold pump fluence, a sharp ASE peak emerges at a longer wavelength than the SPE peak, accompanied by a markedly higher growth rate. Due to the band-filling effect,^43^ the ASE peak exhibits a slight blueshift with increasing pump fluence. Under the highest pump fluence, the ASE peak wavelength of sample S-II (∼803.1 nm) is slightly longer than that of S-I (∼801.5 nm). The pump fluence-dependent evolution of ASE spectra at various temperatures (80–250 K) for S-I and S-II is shown in Figures S11 and S12 (Supporting Information). Figure 3d and e shows the peak intensity as a function of pump fluence for samples S-I and S-II at various operation temperatures (80–300 K). The emission peak intensity and slope efficiency significantly increase when the pump fluence exceeds the threshold, determined from the intersection of two linear fitting lines.

**Figure 3:**
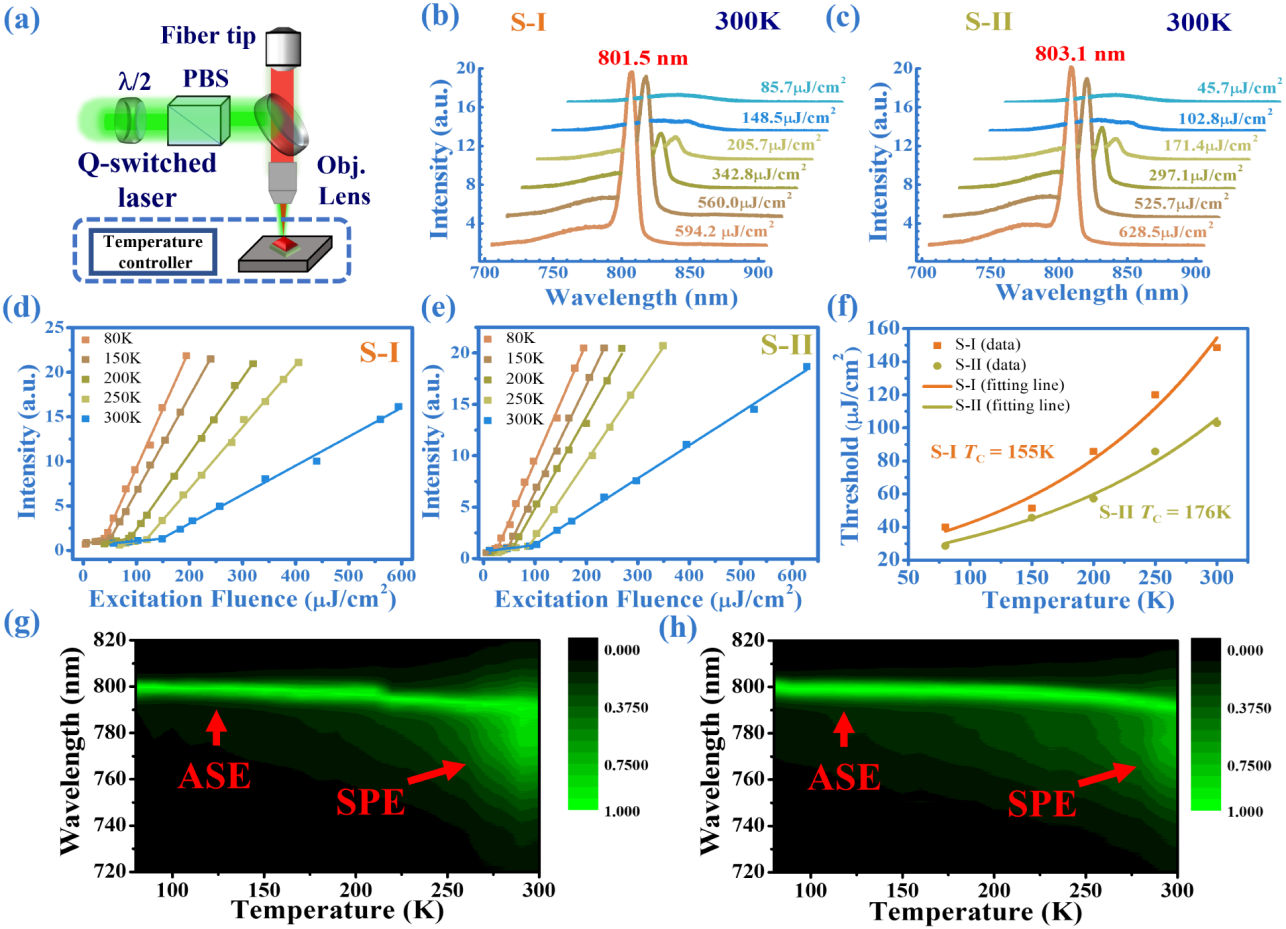
Temperature-dependent ASE measurements of the FAPbI_3_ NC films. (a) Schematic plot showing the experimental setup, evolution of RT ASE spectra with excitation intensity for (b) S-I and (c) S-II, peak intensity versus pump fluence at different temperatures for (d) S-I and (e) S-II, and (f) lasing threshold as a function of temperature (S-I: burnt orange square symbol; S-II: olive green circle symbol; solid line: fitting curve from eq 2 to obtain the characteristic temperature (*T*_C_)). Two-dimensional plots of normalized emission spectra as a function of temperature for samples (g) S-I and (h) S-II.

The lasing thresholds of the two samples as a function of temperature (80–300 K) are shown in Figure 3f. Compared with samples operated at higher temperatures, those operated at lower temperatures exhibited significantly lower thresholds and higher slope efficiencies. At 80 K, the threshold values were 40.0 μJ/cm^2^ and 28.6 μJ/cm^2^ for S-I and S-II, respectively. This reduction is mainly attributed to the suppression of nonradiative recombination and exciton–phonon scattering under low-temperature conditions.^44^ Additionally, the obtained thresholds for sample S-II are lower than those of S-I across all operating temperatures (*T*). The temperature-related threshold variation in Figure 3f can be ascribed by^45^

2 $$E_{\mathrm{th}}\left(T\right)=E_{\mathrm{th},0}\exp\left(\frac{T}{T_{c}}\right)$$

where *E*_th,0_ is the threshold of pump fluence at a low temperature, and *T*_c_ is the characteristic temperature. A good fit to eq 2, as indicated by the burnt orange (S-I) and olive green (S-II) solid lines in Figure 3f, yields characteristic temperatures of approximately 155 and 176 K for S-I and S-II, respectively. The extracted values fall within the range reported for other semiconductor materials, such as CdSe/ZnS quantum rods (∼70 K),^46^ ZnO/ZnMgO MQWs (∼87 K),^47^ and CsPbBr_3_ QDs (∼230 K).^45^ The higher characteristic temperature observed in sample S-II indicates improved thermal stability compared to S-I. This enhancement is associated with the presence of the PMMA layer, which improves film uniformity, passivates interfacial defects, and enhances the mechanical integrity of the perovskite NC film. In addition, the polymer layer acts as a thermal buffering medium that moderates thermally induced local stress and temperature fluctuations.^48,49^ These combined effects help reduce thermally activated nonradiative recombination pathways, thereby contributing to improved stability at elevated temperatures. At a fixed excitation pump fluence (∼140 μJ/cm^2^), the normalized ASE intensity distribution across various temperatures for the two samples are presented in Figure 3g and h. The ASE peak exhibits a blueshift with increasing operating temperature. This behavior can be primarily attributed to lattice thermal expansion,^50,51^ which modifies the band structure and leads to an increase in the effective bandgap in this material system. In contrast to the distinct ASE peak observed in S-II at RT in Figure 3h, the RT ASE feature in S-I is less clear in Figure 3g due to the lower contrast between the ASE peak and the SPE plateau.

### 2.3 RT ASE Properties of FAPbI_3_ NC Films

#### 2.3.1 Sideward-Scattering ASE from FAPbI_3_ NC Films at RT

The RT sideward-scattering ASE of two FAPbI_3_ NC films was investigated under excitation by a 532 nm Q-switched laser, as illustrated by the setup in Figure 4a. A cylindrical lens (CL) with a focal length of 3 cm was used to focus the stripe-shaped pump beam onto the sample. Figure 4b and c shows the evolution of the ASE spectra for samples S-I and S-II as the pump fluence increases. Similar to the previous results, when the pump fluence exceeds the threshold, a sharp and narrow ASE peak appears on top of the SPE plateau. The corresponding peak intensities as a function of pump fluence for the two samples are shown in Figure 4d and e. This result indicates that the slope for S-II (olive green) is significantly larger than that for S-I (burnt orange), and the threshold for S-II is lower than that of S-I. Based on multiple independent measurements of samples with comparable thicknesses and linear fitting of the input–output relationship, the ASE threshold of S-II was determined to be approximately 43.4 ± 3.3 μJ/cm^2^, compared with 98.8 ± 6.3 μJ/cm^2^ for S-I, as summarized in Table 2. The reduced threshold can be attributed to the improved film uniformity in S-II, which mitigates nonradiative recombination losses.

**Figure 4:**
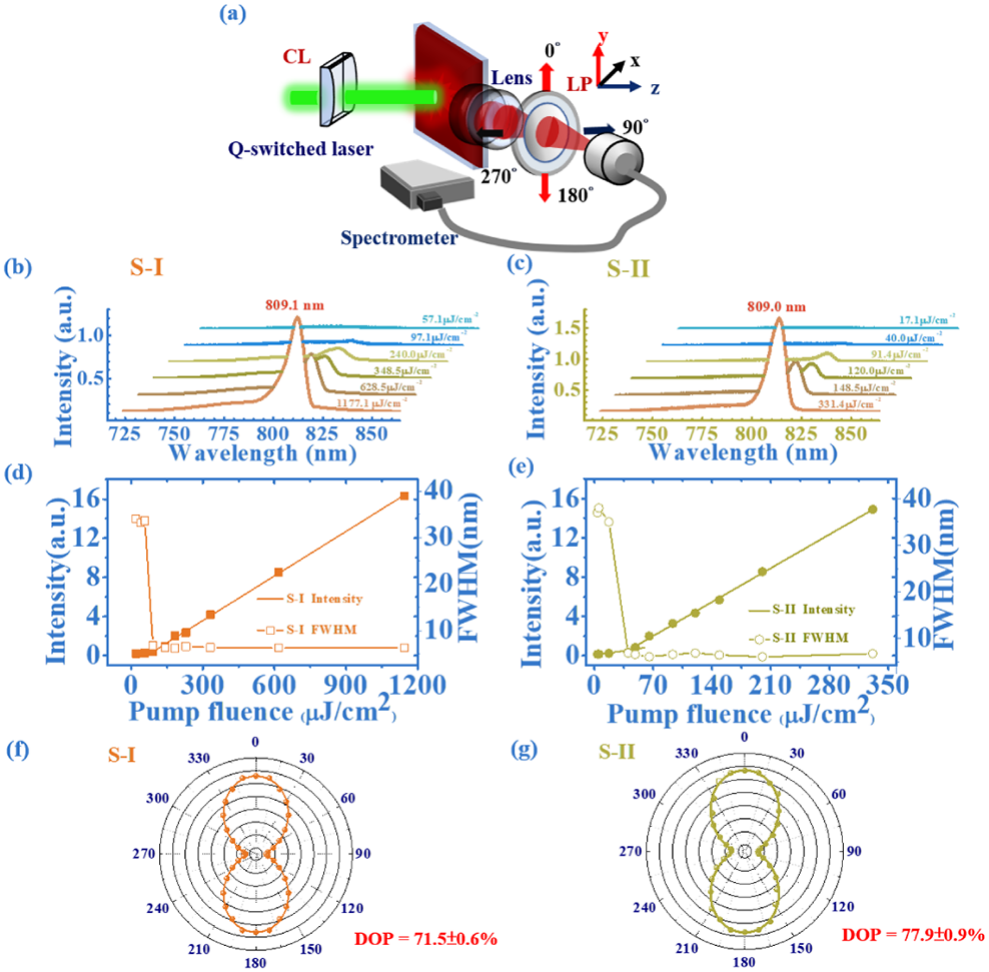
Sideward-scattering ASE characteristics of the FAPbI_3_ NC films. (a) Schematic illustration of the experimental setup. ASE spectral evolution with increasing pump intensity for (b) S-I and (c) S-II, respectively. Peak intensity and FWHM as functions of pump fluence for (d) S-I (burnt orange) and (e) S-II (olive green). Polar plots showing the ASE intensity variation with angle θ for samples (f) S-I and (g)­S-II.

**Table 2:** Comparison of ASE Thresholds and DOP under Sideward-Scattering and Backward-Scattering Configurations for S-I and S-II

	sideward-scattering		backward-scattering	
sample	*E*_th_ (μJ/cm^2^)	DOP (%)	*E*_th_ (μJ/cm^2^)	DOP (%)
S-I	98.8 ± 6.3	71.5 ± 0.6	102.4 ± 6.1	52.5 ± 1.1
S-II	43.4 ± 3.3	77.9 ± 0.9	72.6 ± 3.1	63.9 ± 1.6

Due to the gain-guiding effect,^45^ the linear polarization characteristic has been demonstrated in a CsPbBr_3_ QD film under sideward-scattering measurement. To confirm the polarization characteristic of the FAPbI_3_ NC film, a linear polarizer (LP) was placed in front of the fiber tip, as shown in Figure 4a. The polar plots of ASE intensity as a function of the rotation angle θ of the LP relative to the *y*-axis for the two samples are shown in the Figure 4f and g. A plot showing two side lobes indicates that the sample exhibits partially linear polarization characteristics. Additionally, the DOP was calculated using the following expression: $\mathrm{DOP}=\frac{I_{\max}-I_{\min}}{I_{\max}+I_{\min}}$ , where *I*_max_ and *I*_min_ represent the maximum and minimum ASE intensities, respectively. The estimated DOP value for sample S-II is approximately 77.9 ± 0.9%, which is slightly higher than that of sample S-I (∼71.7 ± 0.6%). In addition to sideward-scattering, we also demonstrated backward-scattering ASE from the FAPbI_3_ NC film using the same CL to focus the pump pulses. The experimental setup, along with the corresponding ASE spectral evolution and polar plot, is provided in Figure S13 (Supporting Information). Compared to sideward-scattering, the backward-scattering configuration exhibits a higher *E*_th_ and a lower DOP for both samples, as listed in Table 2, owing to the weaker gain-guiding effect.

#### 2.3.2 Gain Coefficient Measurement in FAPbI_3_ NC Films

Optical gain in the active medium governs light amplification and is essential for overcoming cavity losses to achieve lasing. Accordingly, a lower lasing threshold typically implies a higher effective optical gain, assuming comparable cavity losses. To quantitatively verify this relationship, the variable stripe length (VSL) method^52,53^ was employed to extract the optical gain coefficient, with the experimental setup illustrated in Figure 5a. In this method, the excitation stripe length is controlled by a movable blade, and the corresponding side-scattered ASE spectra are collected using a spectrometer. Figure 5b presents the ASE peak intensity of samples S-I and S-II as a function of the excitation stripe length under the pump fluence of 360 μJ/cm^2^. As the stripe length exceeds a critical value, the net gain surpasses intrinsic losses, leading to an exponential increase in ASE intensities. With further increases in stripe length, gain saturation occurs and the ASE intensity gradually approaches a plateau. The gain coefficient *g* is obtained by fitting the experimental data using^52–54^

3 $$I\left(L\right)=\frac{A}{g}\left[\exp\left(gL\right)-1\right]$$

where *I*(*L*) is the emission intensity, *L* is the stripe length, and *A* represents the cross-sectional area of the excited region. Based on eq 3, the gain coefficients of S-I and S-II were determined from the fitted curves in Figure 5b to be 140.2 ± 6.9 cm^–1^ and 200.4 ± 8.0 cm^–1^, respectively (S-I: burnt orange; S-II: olive green). Since optical gain depends on the excitation conditions rather than being a fixed constant, the gain coefficients of samples S-I and S-II were extracted at various pump fluences from Figures S14 and S15 (Supporting Information) and summarized in Figure 5c. The data show that S-II exhibits higher optical gain than S-I across the measured excitation range, which is consistent with the lower ASE threshold observed in Figure 4. This behavior is attributed to the underlying PMMA layer, which passivates interfacial defects and improves both the morphological and optical quality of the perovskite NC film, thereby reducing nonradiative recombination losses. As a result, sample S-II exhibits a lower ASE threshold, an enhanced gain coefficient, and a higher degree of polarization than the FAPbI_3_ NC film without the PMMA buffer layer.

**Figure 5:**
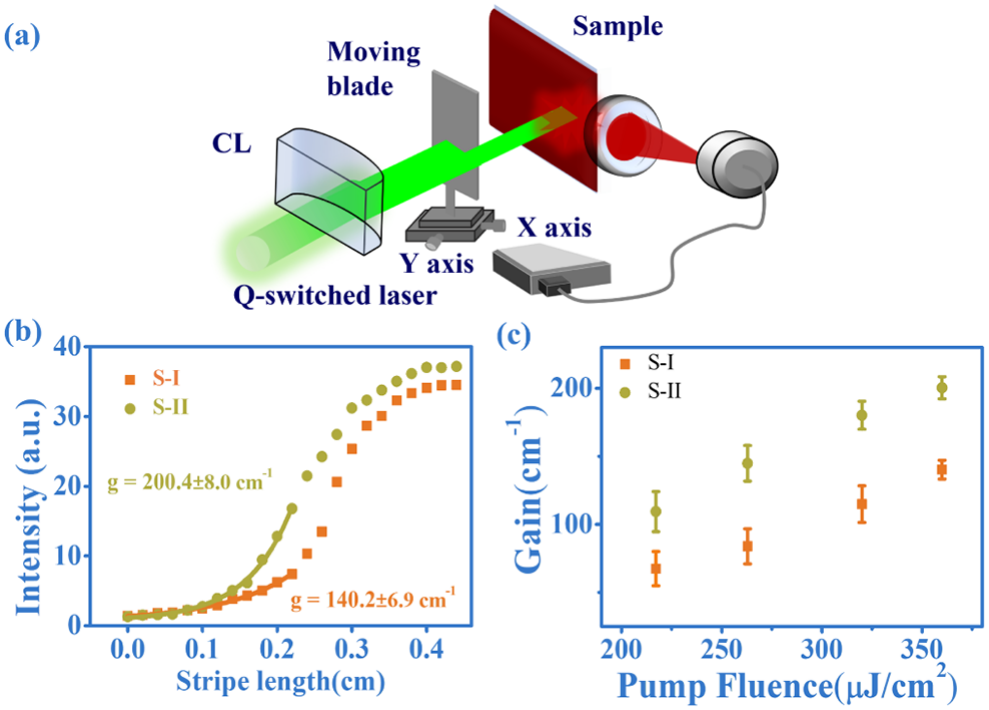
Gain coefficient measurement of the FAPbI_3_ NC films via VSL technique. (a) Schematic illustration of the experimental setup. (b) ASE peak intensity as a function of stripe length (S-I: burnt orange solid squares, S-II: olive green solid circles, solid curves: theoretical fittings using eq 3). (c) Pump fluence dependence of the gain coefficients for S-I and S-II. Error bars indicate the standard error of the fitting parameters.

#### 2.3.3 ASE Tunability on Flexible PET Substrates

Owing to their potential applications in flexible displays and wearable sensors, flexible organic and inorganic semiconductor lasers^55,56^ have attracted considerable attention. Notably, key laser parameters, including the emission wavelength, output intensity, and lasing threshold, can be tuned via mechanical bending.^25,56^ Consequently, various flexible substrates have been explored as alternatives to conventional rigid glass substrates. For example, CsPbBr_3_ QD films were deposited on SF films, with flexible polyethylene terephthalate (PET) serving as the substrate.^25,56^ Other reports have demonstrated that increasing bending strain enhances the random lasing intensity of MAPbBr_3_ thin films on polyimide (PI) substrates,^57^ while mechanical deformation enables the transfer of incoherent random lasing of the CsPbBr_3_ QD film on nickel foam to ASE.^58^ However, the flexible ASE behavior of FAPbI_3_ NC films has rarely been investigated.

To explore the flexible ASE characteristics of the FAPbI_3_ NC film/PMMA composite layer, a bendable polyethylene terephthalate (PET) substrate was employed in place of conventional glass substrates, and the resulting sample was denoted as S-II/PET. As shown in Figure 6a, the S-II/PET sample was mounted on a precision-adjustable bending fixture during ASE measurements and subjected to controlled mechanical deformation from the concave state (*C* < 0) to the convex state (*C* > 0). The bottom of Figure 6a displays photographs of the sample under two distinct bending states. Figure 6b presents the evolution of the ASE spectra during the bending process, revealing changes in both emission peak wavelength and intensity with varying curvature. The dependence of the ASE peak wavelength (blue square line) and intensity (red circle line) on curvature is shown in Figure 6c. Notably, the ASE peak wavelength exhibits a red shift from 806.9 to 809.4 nm as the curvature increases from −0.91 cm^–1^ (concave) to +0.91 cm^–1^ (convex). In previous reports,^59,60^ the energy of the *N*th longitudinal mode can be expressed as $E_{N}=\frac{hc}{\lambda_{N}}=\frac{hc}{2L}\frac{N}{n\left(\omega \right)}$ , where *h* is Planck’s constant, *L* is the effective cavity length, and *n*(ω) is the refractive index. Accordingly, the observed redshift may be associated with a slight increase in the refractive index as the FAPbI_3_ NC film transitions from a concave to a convex state. The ASE intensity increases with the degree of bending deformation in both concave and convex states. The increase in intensity under both conditions may be attributed to enhanced light scattering, as further confirmed by PL measurements conducted with a similar setup excitation with a CW He–Cd laser. The measured results shown in Figure 6d and e exhibit a similar trend.

**Figure 6:**
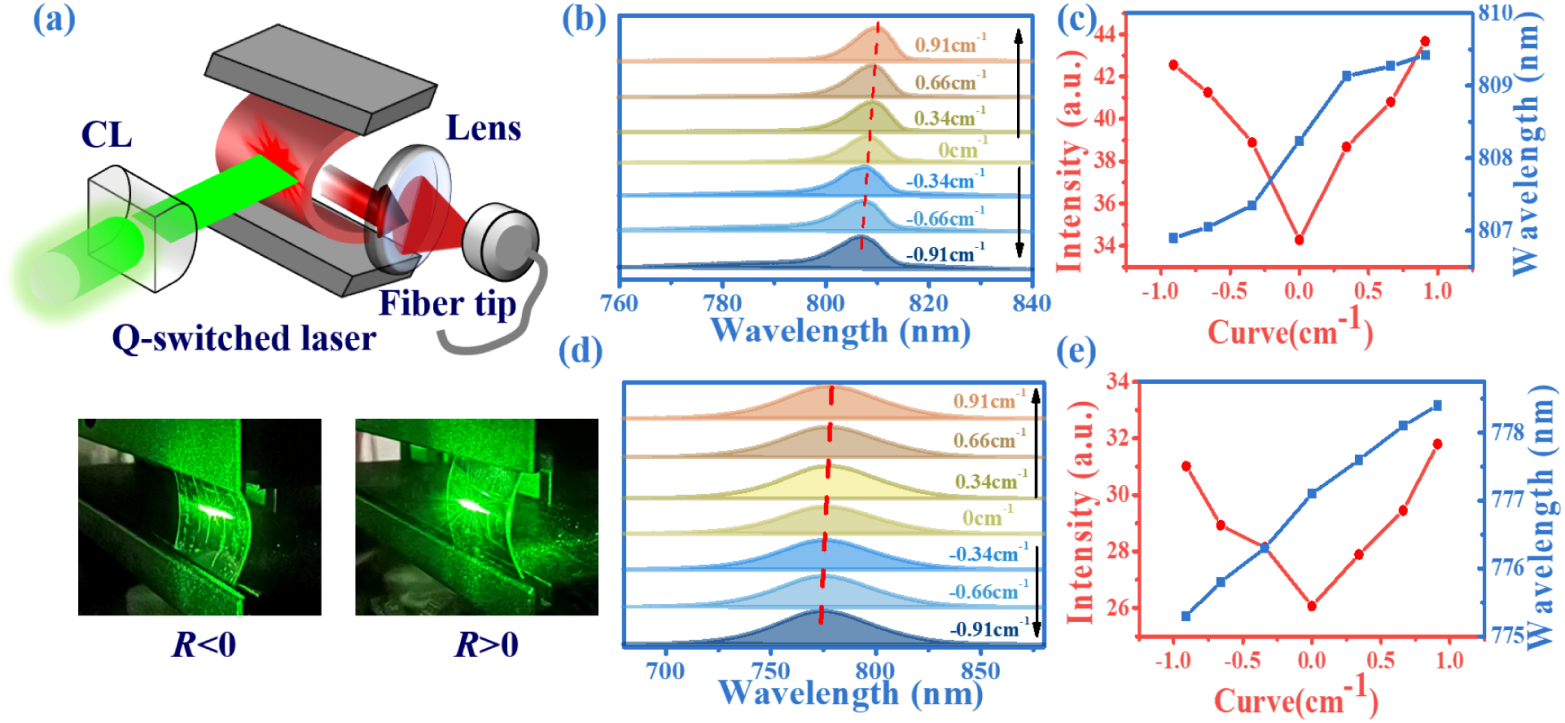
ASE characteristics of a flexible FAPbI_3_ NC film/PMMA structure on a PET substrate (S-II/PET) under mechanical stress. (a) Schematic illustration of the experimental setup (top) and a corresponding photograph showing the bent sample in concave (*R* < 0) and convex (*R* > 0) states, (b) evolution of ASE spectra as the sample was bent from the concave state to the convex state, (c) corresponding ASE peak intensity and wavelength as a function of curvature, (d) evolution of PL spectra as the sample was bent from the concave state to the convex state, and (e) corresponding PL peak intensity and wavelength as a function of curvature.

In addition, sample S-III/PET was fabricated in which the FAPbI_3_ NC layer was encapsulated between two PMMA layers to construct a symmetric sandwich structure. Similar to the behavior observed in sample S-II/PET, S-III/PET exhibits more pronounced wavelength shifts and intensity enhancements under bending, as evidenced by the spectral evolution and the corresponding variations in peak wavelength and intensity shown in Figure S16 (Supporting Information). Compared with S-II/PET, both the PL and ASE of S-III/PET show more significant wavelength shifts and greater intensity enhancements, which can be attributed to the stronger waveguiding effect provided by the double-layer PMMA encapsulation.

## 3 Conclusions

The photophysical properties, including ASE and 2PA of the FAPbI_3_ NC film assisted by the PMMA layer, were investigated in this work. In comparison to the sample directly deposited on a glass substrate, the PMMA-assisted FAPbI_3_ NC film reveals better morphology and film quality, resulting in higher-efficiency PL emissions in both one-photon and two-photon absorption processes. Through temperature-dependent ASE measurements, sample S-II exhibits a higher characteristic temperature (∼176 K) than S-I (∼155 K), indicating higher thermal stability. The low-threshold ASE behavior of both NC films was demonstrated under ns-pulse excitation from a Q-switched laser at RT, in both backward-scattering and sideward-scattering configurations. Under the sideward-scattering configuration, the PMMA-assisted NC film (S-II) reveals lower threshold ASE (43.4 ± 3.3 μJ/cm^2^), higher slope efficiency, gain coefficient and degree of polarization (∼77.9 ± 0.9%) than that without PMMA (S-I) owing to the passivation by the PMMA layer. Due to the lateral waveguide effect, the performance in the sideward-scattering configuration is better than backward-scattering configuration. By adopting a bendable PET as substrate, the flexible lasing behavior of FAPbI_3_ NC film was demonstrated, which shows obvious wavelength tuning and intensity modulation as curvature variation. These findings highlight the potential of PMMA-assisted FAPbI_3_ NC films for application in next-generation laser and high-performance flexible optoelectronic devices.

## 4 Experimental Section

### 4.1 Sample Preparation

#### 4.1.1 Materials

Chemicals such as formamidinium iodide (FAI), lead iodide (PbI_2_), 1-octadecene (ODE), oleic acid (OA), anhydrous *n*-hexane (C_6_H_14_), cyclohexane (C_6_H_12_), anhydrous ethyl acetate (C_4_H_8_O_2_), and octadec-9-enylamine (OAm) were purchased from Sigma-Aldrich.

#### 4.1.2 Preparation of FAPbI_3_ NCs

No further purification was performed on the chemicals. The FAPbI_3_ NCs were synthesized by the hot-injection method. First, precursor-I (P–I) was produced by adding OA (5 mL) solution to a three-necked flask and heated to 80^◦^C under an Ar atmosphere with stirring using a magnetic stir bar. Then, the mechanical pump was activated to create a vacuum environment inside the flask. Subsequently, FAI powder was added and dissolved completely into the OA solution. To prepare precursor-II (P–II), ODE (10 mL), OA (2 mL), and OAM (1.3 mL) were added into another three-necked flask and heated to 120 °C under an Ar atmosphere with stirring. Then, PbI_2_ powder was added to the solution under vacuum in the flask. Next, 2 mL of the preheated FAI solution (P–I) at 80 °C was rapidly injected into P–II, confirming that the mixture turned dark red. Finally, the three-necked flask was quickly immersed in an ice bath to stop the reaction.

Ligand treatment was used for the purification of FAPbI_3_ NCs and for removing excess ligands. First, the synthesized mixture was centrifuged at 10,000 rpm for 10 min to precipitate the FAPbI_3_ NCs at the bottom of the centrifuge tube. The collected precipitated NCs were mixed with anhydrous *n*-hexane and anhydrous ethyl acetate in a 1:1 ratio for two additional centrifugation steps to remove excess ligands and impurities. Cyclohexane was added to the purified FAPbI_3_ NCs for centrifugation. Finally, the collected supernatant was filtered using a 0.22 μm filter to obtain the small-sized FAPbI_3_ NCs.

#### 4.1.3 Fabrication of FAPbI_3_ NC Films

Two different FAPbI_3_ NC films, labeled S-I and S-II, were fabricated. For sample S-I, the pure FAPbI_3_ NC film was prepared by directly spin-coating the dispersed solution onto a glass substrate at 2000 rpm for 30 s. For sample S-II (PMMA/FAPbI_3_ NC film), the PMMA solution was first spin-coated onto a glass substrate at 3000 rpm, and after drying, the FAPbI_3_ NC solution was spin-coated on top.

### 4.2 Optical Setup

#### 4.2.1 One-Photon Absorption PL Measurements

A continuous-wave He–Cd laser (IK3301R-G, KIMMON KOHA Inc.) with a wavelength of 325 nm was used as the pump source. After being introduced into the microscope system, the pump beam was reflected by an edge filter (SP01–355RU-25, Semrock Inc.) and then focused onto the sample through a 10× objective lens (LMU-10X-NUV, Thorlabs Inc.). The PL emission from the sample was collected by the objective lens, transmitted through the edge filter once again, and then collected by a fiber tip. Finally, the emission spectrum was measured by a monochromator (iHR320, Horiba Inc.) equipped with a charge-coupled device (CCD) (Syncerity, Horiba Inc.).

#### 4.2.2 Two-Photon Absorption PL Measurement

A passively mode-locked ytterbium-doped fiber laser (Kasmoro-1070, mRadian Inc.) with a wavelength of 1070 nm was used as the excitation source. The laser beam was first reflected by a beam splitter (DMSP1000, Thorlabs Inc.) and then focused onto the sample using a 5× objective lens (M Plan 5*X*/0.10, Nikon Corp.) with a focal length of 40 mm. The emission signal emitted from the sample was collected by the same objective lens, passed through the beam splitter again, and then expanded by two lenses before being delivered into a fiber probe. Finally, the emission spectra were recorded using a monochromator (iHR320, Horiba Inc.) equipped with a CCD detector.

#### 4.2.3 Temperature-Dependent ASE Measurements

A frequency-doubled Q-switched Nd:YAG laser with a central wavelength of 532 nm, a pulse duration of 2.4 ns, and a repetition rate of 10 Hz was used as the light source. To control the pump fluence, a polarization beam splitter (PBS) and a half-wave plate (λ/2) were inserted into the optical path. The sample was mounted in a cryostat (ST-500-UC, JANIS Inc.), and the temperature was controlled using a temperature controller (Model 335, Lake Shore Inc.). The pump beam was directed into the microscopy system, passed through a long-pass filter (LP03–532RE-25, Semrock Inc.), and was then focused onto the sample by a 10× objective lens. The ASE spectra from the surface normal of the sample were collected by the same objective lens and finally recorded using a monochromator (iHR550, Horiba Inc.) equipped with a CCD detector (Syncerity, Horiba Inc.).

#### 4.2.4 RT ASE Measurements

A frequency-doubled, Q-switched Nd:YAG laser with a central wavelength of 532 nm, a pulse duration of 2.4 ns, and a repetition rate of 10 Hz was used as the light source.

##### 4.2.4.1 Sideward ASE Measurements

The pump beam was focused onto the sample with a long stripe (∼ 3.3 mm × 0.95 mm) through a CL with a focal length of about 3 cm. The emission coming out from the edge of the sample was collected into a fiber tip by a pair of lenses. The ASE spectrum was recorded using a monochromator (iHR550, Horiba Inc.) equipped with a CCD detector (Syncerity, Horiba Inc.).

##### 4.2.4.2 Polarization Measurements

To investigate the characteristics, a linear polarizer was placed in front of the fiber tip.

##### 4.2.4.3 Flexible ASE Measurements

The sample was fixed onto a custom-built mechanical loading fixture designed to apply a controlled stress. The fixture itself was mounted on a high-precision three-dimensional translation stage, allowing fine adjustment along the x-, y-, and *z*-directions and ensuring consistent alignment with the excitation stripe and the collection optics throughout the deformation process.

## Supplementary Material



## Notes

Thickness measurement; XPS spectra; EDS images; Tauc plot; PL mapping; time-resolved PL decay curves; two-photon absorption PL spectra; optical limiting measurements; temperature-dependent ASE measurement; backward scattering ASE measurement; gain coefficient measurement; and flexible ASE measurement (PDF)
