# Supplementary material for: Amplified Spontaneous Emission Enhancement in FAPbI3 Nanocrystal Films via PMMA and Mechanical Tunability on Flexible PET
Source: ACS Appl Mater Interfaces. 2026 May 21;18(21):30411–21. doi: 10.1021/acsami.6c05503 (PMC13244371; doi:10.1021/acsami.6c05503)
Supplement: Supplementary file 1 [file am6c05503_si_001.pdf]

Supporting Information

Amplified Spontaneous Emission Enhancement in $FAPbI_3$ Nanocrystal Films via PMMA and Mechanical Tunability on Flexible PET

Ja-Hon Lin,*,† Yu-Ming Li,† Kuan Yu Liao,† Chun-Chung Chen,† Hsin-Yi Wei,† Kai-Wei Lin,‡ Chi-Ching Kuo,*,‡ Bi-Hsuan Lin,¶ Youhei Chitose, §,‖,⊥ and Chihaya Adachi*,§,‖

J.-H. Lin, Y.-M. Li, K.-Y Liao, C. -C. Chen, H. -Y. Wei

† Department of Electro-Optical Engineering, National Taipei University of Technology,Taipei 106, Taiwan

‡ Institute of Organic and Polymeric Materials, National Taipei University of Technology,Taipei 106, Taiwan

¶ TPS 23A X-ray Nanoprobe Beamline, National Synchrotron Radiation Research Center,Hsinchu 30076, Taiwan

§Center for Organic Photonics and Electronics Research (OPERA) Kyushu University, 15Fukuoka, Fukuoka 819-0395, Japan

‖ International Institute for Carbon Neutral Energy Research (I2CNER), Kyushu University, 744 Motooka, Nishi, Fukuoka 819-0395, Japan

⊥ Department of Applied Chemistry, Graduate School of Engineering, Center for Molecular Systems(CMS), Kyushu University, Fukuoka 819-0395,Japan

E-mail: jhlin@ntut.edu.tw; kuocc@ntut.edu.tw; adachi@cstf.kyushu-u.ac.jp

Thickness of $FAPbI_3$ NC films

The thickness of the $FAPbI_3$ NC films was measured using a DEKTAK surface profiler. Step-height profiles obtained from the film edge were used to determine the film thickness, as shown in Figures S1a and S1b. The green shaded regions indicate the selected ranges for averaging the thickness values. Based on measurements performed at five different locations on each sample, the average film thicknesses were determined to be approximately 544.6 and 541.6 nm for samples S-I and S-II, respectively, as summarized in Table S1.

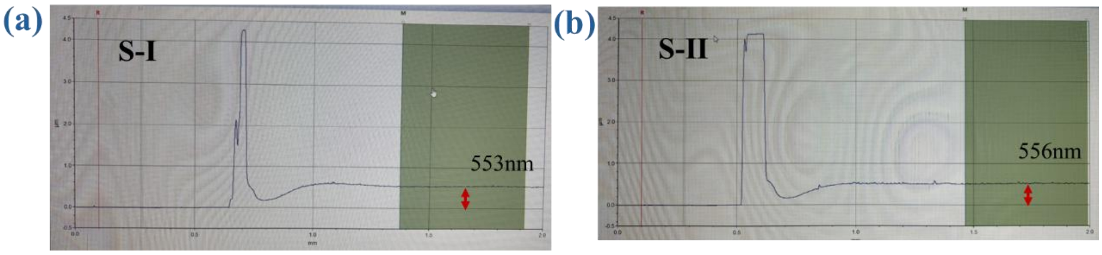

Figure S1. Step-height profiles used to determine the thicknesses of the $FAPbI_3$ NC films: (a) sample S-I (~544.6 nm) and (b) sample S-II (~541.6 nm).

Table S1 Film thickness of two samples measured at different locations and average thickness values.

	L-I (nm)	L-II (nm)	L-III (nm)	L-IV (nm)	L-V (nm)	Ave. (nm)
S-I	552	551	528	539	553	544.6
S-II	552	529	520	556	551	541.6

XPS spectra of $FAPbI_3$ NC films

Figure S2 shows the XPS binding energy spectra of I, Pb, C, and O for two samples. The I $3d_{5/2}$ and $3d_{3/2}$ peaks are observed at approximately 618.2 eV and 629.7 eV, respectively; the Pb $4f_{7/2}$ and $4f_{5/2}$ peaks appear near 137.3 eV and 142.1 eV, and the C 1s peak is located around 284 eV. Additionally, an O 1s signal appears near 531.5 eV, which is likely due to oxidation upon exposure to air. These binding energies closely

match the characteristic energy levels of each element in the $FAPbI_3$ structure, indicating stable chemical states and consistent with previously reported results. Moreover, no significant spectral shifts were observed in the S-II sample, suggesting that the addition of PMMA does not alter the chemical structure, which is also in agreement with prior results[1].

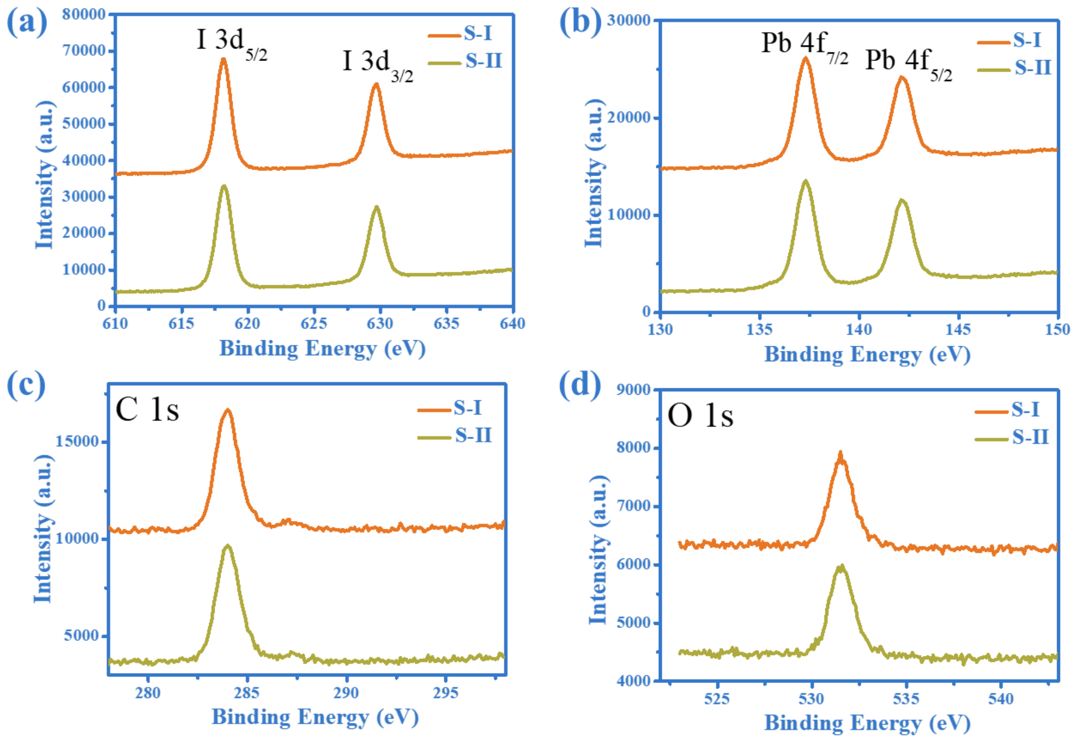

Figure S2. Fine XPS spectra of $FAPbI_3$ NC films for the element: (a) I 3d, (b) Pb 4f, (c) C 1s, (d) O 1s.

EDS images of $FAPbI_3$ NC films

The chemical composition of two $FAPbI_3$ NC films was confirmed using energy-dispersive X-ray spectroscopy (EDS), as shown in Figures S3(a) for the S-I and Figure S3(b) for the S-II. $FAPbI_3$ NCs belong to the organic-inorganic perovskite and are primarily composed of elements such as carbon, hydrogen, and nitrogen. Since hydrogen cannot be detected by EDS, only the atomic ratio between lead (Pb) and iodine (I) was analyzed. The measured Pb:I ratio is approximately 1:3, which is consistent with the theoretical stoichiometric ratio of $FAPbI_3$. Elemental mapping of lead, and iodine shown in right hand side of Figures S3(a) and (b) reveals a uniform distribution of these elements in both the S-I and S-II samples.

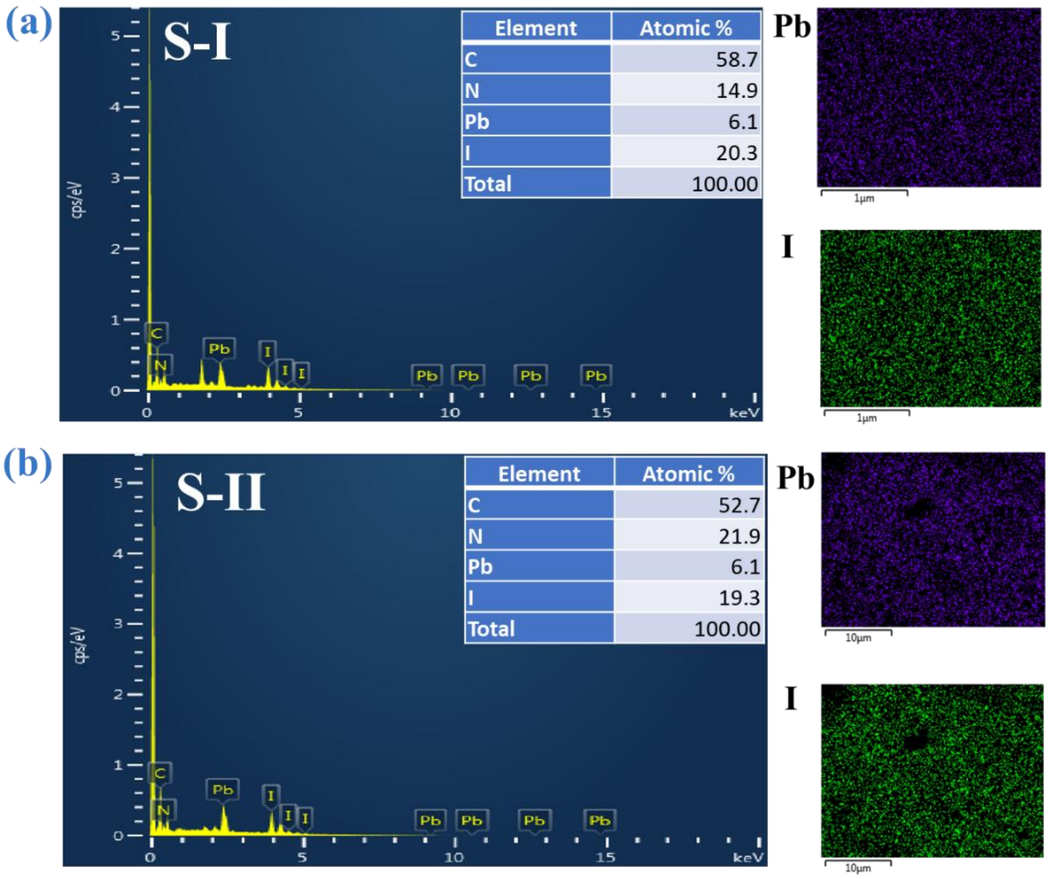

Figure S3 EDS image (left) of $FAPbI_3$ NC film and the mapping (right) of Pb and I for (a) S-I, and (b) S-II.

Tauc plot of $FAPbI_3$ NC films

The Tauc plots of samples S-I and S-II, derived from their absorption spectra, are shown in Figures S4(a) and (b), respectively. These plots are used to determine the optical bandgaps based on the following equation:

$$(\alpha h\nu)^2 = A(h\nu - E_g), \qquad (1)$$

where α represents the absorption coefficient, $h\nu$ is the photon energy, E_g is the optical bandgap, A is a constant. Based on Eq. (1) in the supporting information, both samples exhibit a bandgap of approximately 1.62 eV (765 nm), which aligns closely with the reported bandgap of $FAPbI_3$ NC thin films (~1.63 eV).[2].

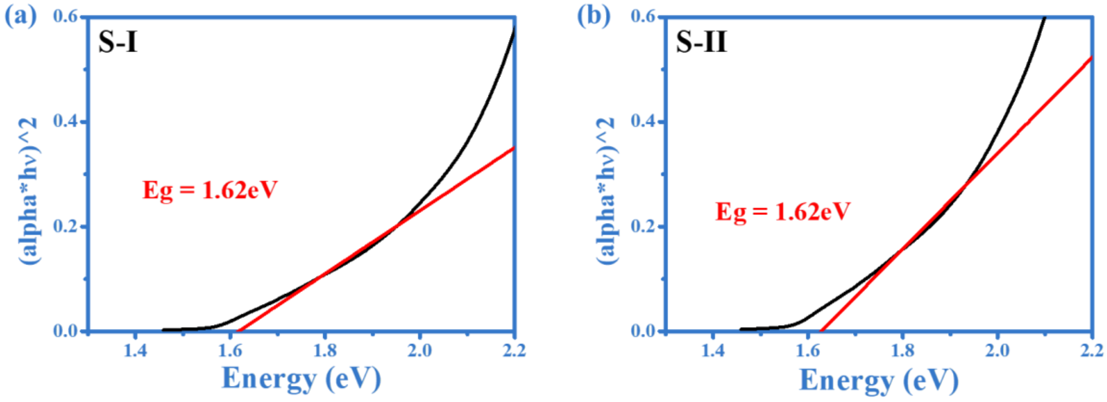

Figure S4. Tauc plot of $FAPbI_3$ NC film for (a) S-I and (b) S-II.

PL Mapping of $FAPbI_3$ NC films

To further evaluate the spatial uniformity of the $FAPbI_3$ NC films, PL mapping was performed using a 325 nm CW He-Cd laser as the excitation source. Figures S5(a) and S5(c) show the 2D PL peak wavelength distribution for samples S-I and S-II, respectively, where the color contrast reflects spatial variations in emission wavelength. For quantitative analysis, the 2D PL data were processed to generate histograms of the overall PL intensity distributions, as shown in Figures S5(b) and S5(d). Similar, Figures S6(a) and S6(c) present the 2D FWHM distribution for samples S-I and S-II, respectively, where the color contrast indicates the spatial variation in FWHM. The corresponding histograms of the overall FWHM distributions are shown in Figures S6(b) and S6(d). From the histograms, it can be observed that sample S-II exhibits significantly narrower distributions in both PL peak wavelength and FWHM compared to sample S-I, indicating improved spectral uniformity.

This improvement is attributed to the effective passivation of surface defects in $FAPbI_3$ NCs by the PMMA layer. Surface defects perturb the local band structure and introduce energetic disorder, which broadens the distribution of transition energies within the nanocrystal ensemble. This defect-induced disorder contributes primarily to inhomogeneous broadening, leading to variations in both the PL peak position and the FWHM across different regions of the film. Consequently, spatial variations in defect density result in local spectral fluctuations, where different regions emit at slightly different photon energies and linewidths. In this work, the PMMA layer functions as an effective surface passivation layer that reduces surface trap states and suppresses defect-induced energetic disorder. As a result, the optical uniformity of the $FAPbI_3$ NC film is improved, leading to reduced spatial variations in both the PL peak wavelength and the FWHM across the film.

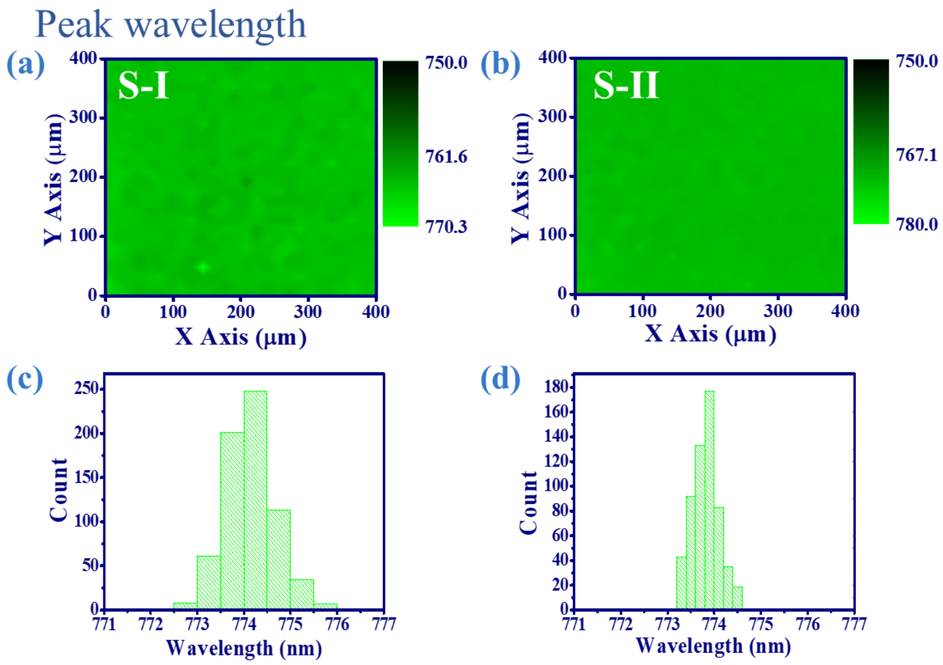

Figure S5 2D PL mapping of the $FAPbI_3$ NC films showing the peak position distribution for (a) S-I and (b) S-II, along with the corresponding histograms for (c) S-I and (d) S-II.

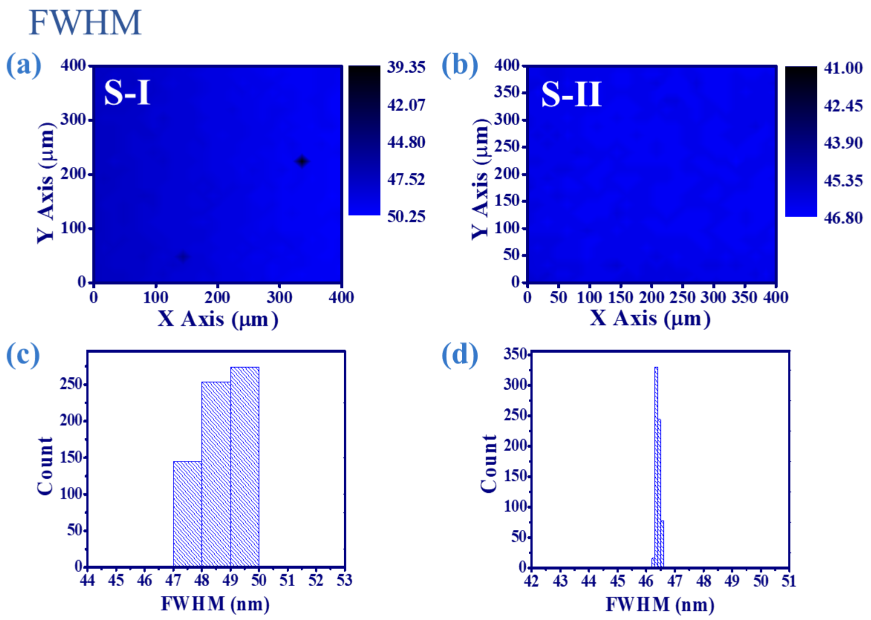

Figure S6. 2D PL mapping of the $FAPbI_3$ NC films showing the FWHM distribution for (a) S-I and (b) S-II, along with the corresponding histograms for (c) S-I and (d) S-II.

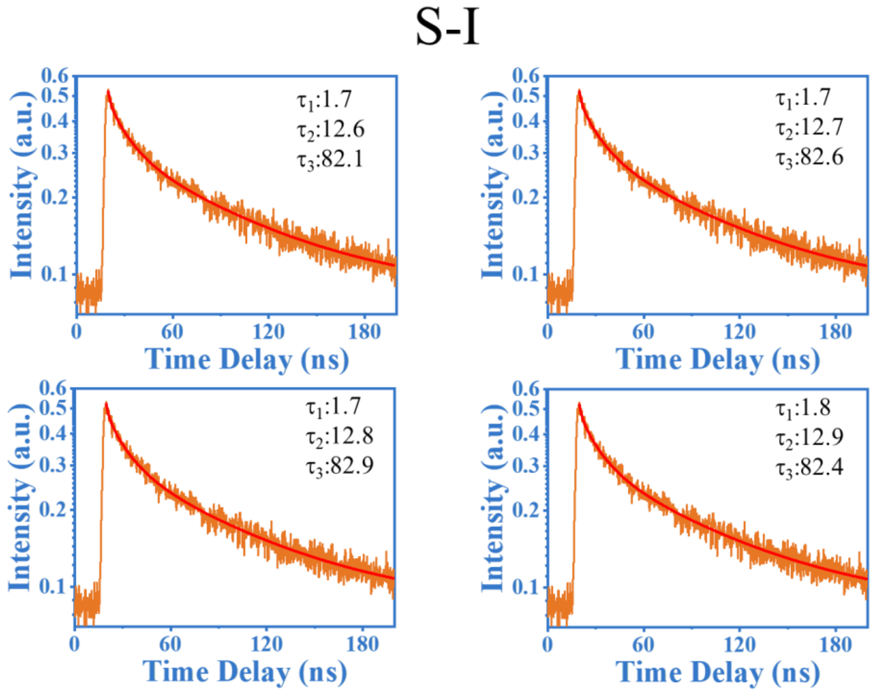

Figure S7 TRPL decay curves of sample S-I. Represent the TRPL results measured at four different positions on the sample.

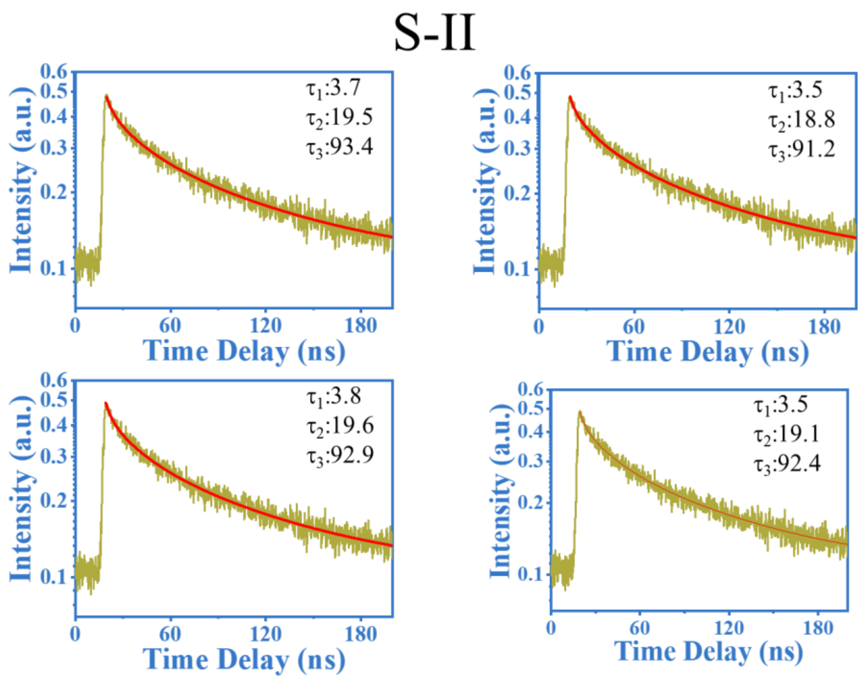

Figure S8 TRPL decay curves of sample S-II. Represent the TRPL results measured at four different positions on the sample.

TPA PL of FAPbI3 NC films

Figures S9(a) and S9(b) show the evolution of TPA PL spectra with increasing peak power of a 1070 nm pulsed laser for samples S-I and S-II, respectively. The TPA PL emission peaks are located at 776.3 nm for S-I and 776.6 nm for S-II.

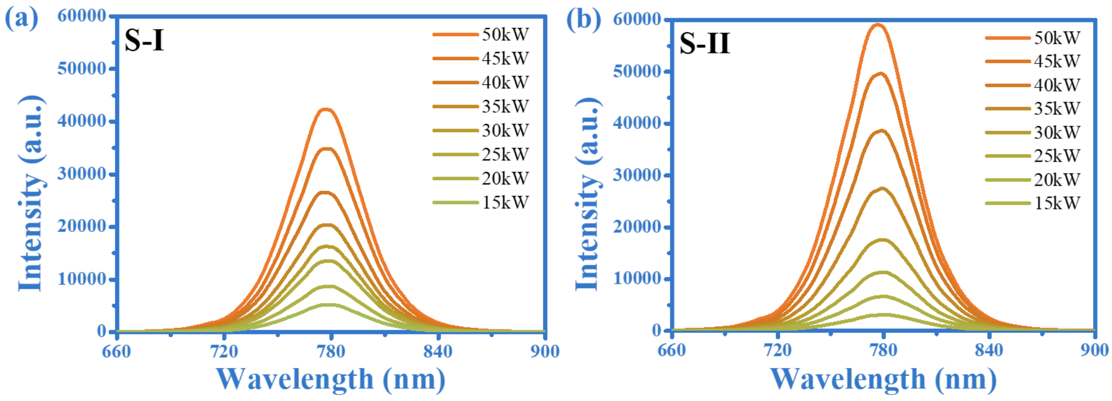

Figure S9. Evolution of TPA-PL spectra of $FAPbI_3$ NC films with increasing peak power for (a) S-I and (b) S-II.

Optical limiting measurement

Figure S10(a) illustrates the experimental setup used to demonstrate optical limiting, in which a PML Yb-doped fiber laser with a central wavelength of approximately 1070 nm and a pulse duration of approximately 150 fs was employed as the excitation source. After being divided by the beam splitter (BS), the reflected light was detected by detector D_1 as a reference beam. By means of a focusing lens with a focal length of f = 4.5 cm, the beam transmitted through the sample was detected by detector D_2. The relationship between the input and output power for ultrashortpulsed light passing through the two samples (S-I and S-II) is shown in Figure S10 (b). At low input powers, the output power exhibits a linear relationship; however, at higher powers, the curves deviate from the linear dashed line due to the TPA effect. In comparison, the output power of sample S-II exhibits a greater deviation and reduction under the same input conditions owing to the stronger TPA effect. The more pronounced deviation from

linearity observed in S-II indicates a stronger two-photon absorption (TPA) induced optical limiting effect compared to S-I.

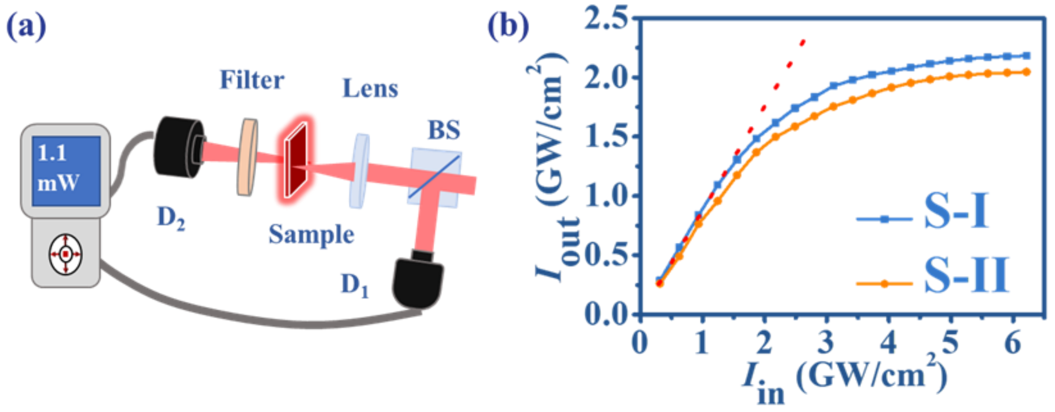

Figure S10. Optical limiting properties of the $FAPbI_3$ NC films measured under 1070 nm fs pulsed laser excitation. (a) Schematic illustration of the experimental setup.(b) Output peak intensity (I_{out}) as a function of input peak intensity (I_{in}) for samples S-I (blue squares) and S-II (orange circles). The red dashed line represents the linear transmission regime.

Temperature-dependent ASE measurement

To investigate the temperature-dependent behavior of ASE, power-dependent ASE measurements were conducted on samples S-I and S-II at various temperatures, as shown in Figures S11 and S12.

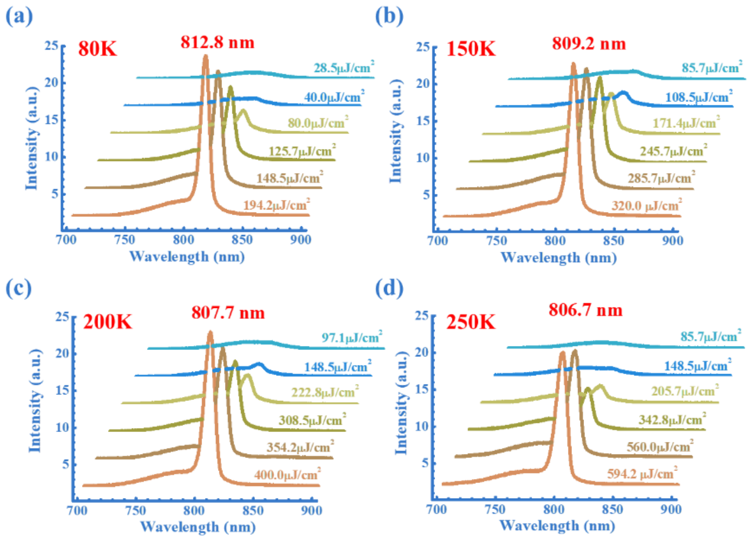

Figure S11 Pump fluence-dependent ASE evolution of S-I at (a) 80 K, (b) 150 K, (c) 200 K, and (d) 250K.

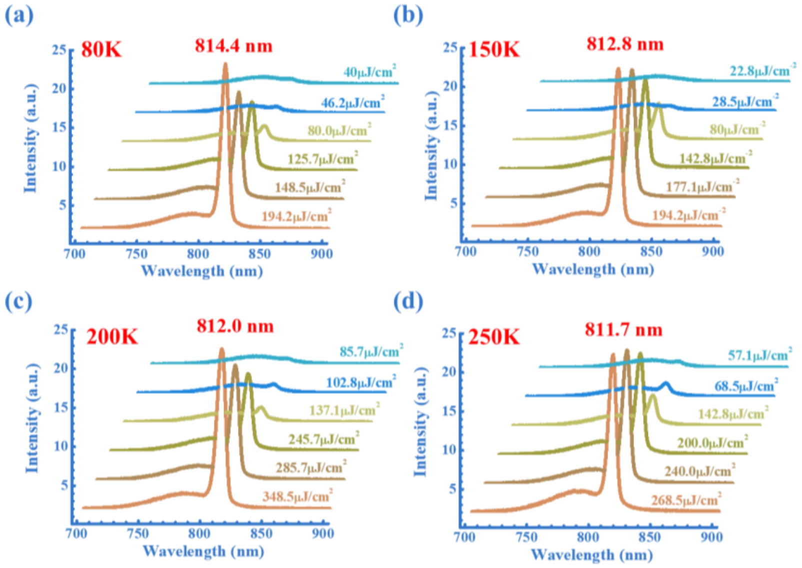

Figure S12 Pump fluence-dependent ASE evolution of S-II at (a) 80 K, (b) 150 K, (c) 200 K, and (d) 250K.

Backward scattering ASE measurement

Figure S13(a) shows the setup for the backward ASE measurement under an excitation by the 532 nm Q-switched laser. Similar to the sideward scattering configuration, the incident puled light is refracted by a dichroic beam splitter (BS) and then focused onto sample by a cylindrical lens (CL) with a focal length of 3 cm. The generated ASE from surface of sample was collected by a CL and then passes through the dichroic BS. After collection by a fiber tip and then measured by a spectrometer. The corresponding ASE peak intensity versus pump fluence is shown on the right side of Figure S13(a), indicating that the threshold for S-II (~74.2 μJ/cm²) is significantly lower than that of S-I (~102.8 μJ/cm²).

Figures S13(b) and (c) show the evolution of ASE spectra for samples S-I and S-II, respectively, with increasing pump fluence. When the pump fluence exceeds the threshold, a sharp and narrow ASE peak emerges atop the spontaneous emission background. At the highest pump fluence, the ASE peaks are located at approximately 810.8 nm for S-I and 810.9 nm for S-II. The insets of Figures S13(b) and (c) show polar plots of ASE intensity as a function of the rotation angle (θ) of the linear polarizer (LP) relative to the y-axis. Consistent with the sideward scattering measurements, the estimated degree of polarization (DOP) for S-II (~64.7%) is higher than that of S-I (~52.3%). This observation aligns with the findings from our side scattering measurements, further confirming the lower trap density and smoother film morphology of S-II.

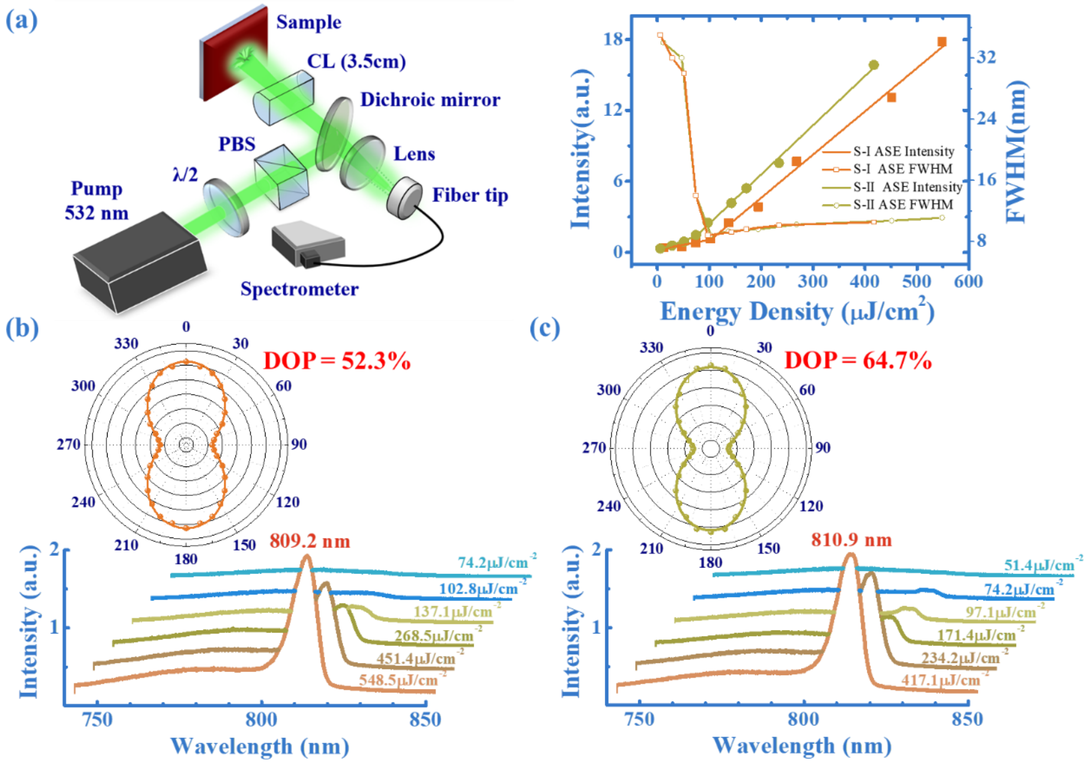

Figure S13. Backward scattering measurement of $FAPbI_3$ NC films. (a) Schematic illustration of the experimental setup (left-hand side), along with the intensity and FWHM as functions of pump fluence for samples S-I (burnt orange) and S-II (olive green) (right-hand side). ASE spectral evolution with increasing pump fluence for (b) S-I and (c) S-II. (Insets: Polar plots showing intensity variation with angle θ.)

Gain coefficient measurement of the $FAPbI_3$ NC film.

Here, the gain coefficient of $FAPbI_3$ NC film is measured by the VSL method by the experimental setup shown in Figure 5(a). Figure S15(a)-(d) and Figure S14(a)-(d) show the ASE peak intensity of samples S-I and S-II as a function of pump stripe length at pump fluences of 217.1, 262.9, 320, and 360μJ/cm^2 respectively. Using Eq. (3) in the main manuscript, the gain coefficients (g) of samples S-I and S-II were derived and are labeled in each plot. The comparison demonstrates that S-II exhibits consistently higher optical gain than S-I under identical excitation conditions.

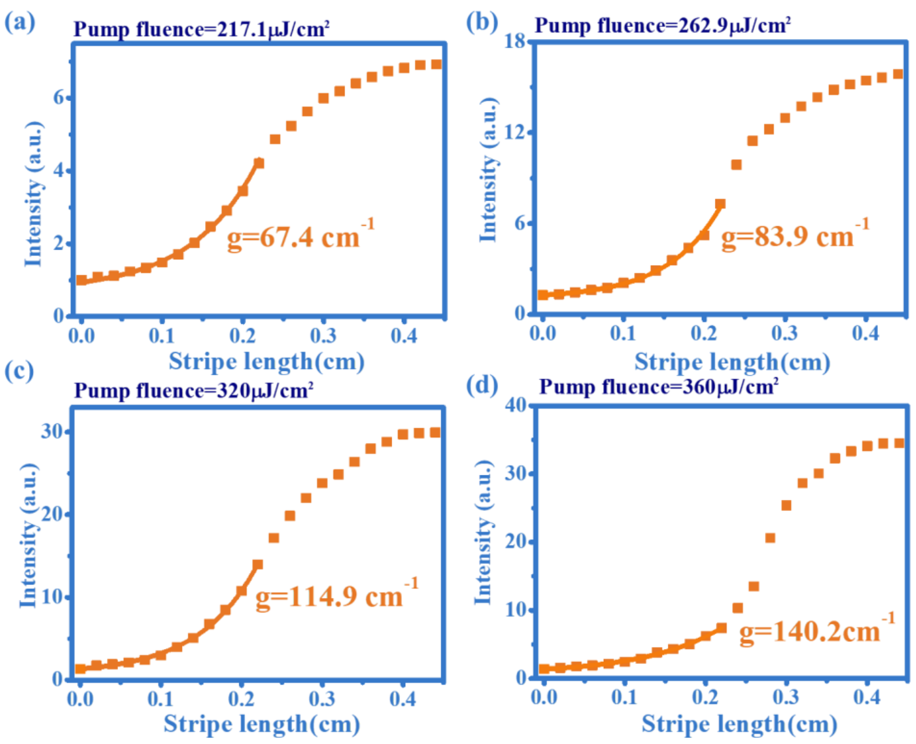

Figure S14 ASE peak intensity as a function of stripe length for sample S-I under excitation pump fluence of (a)-(d)

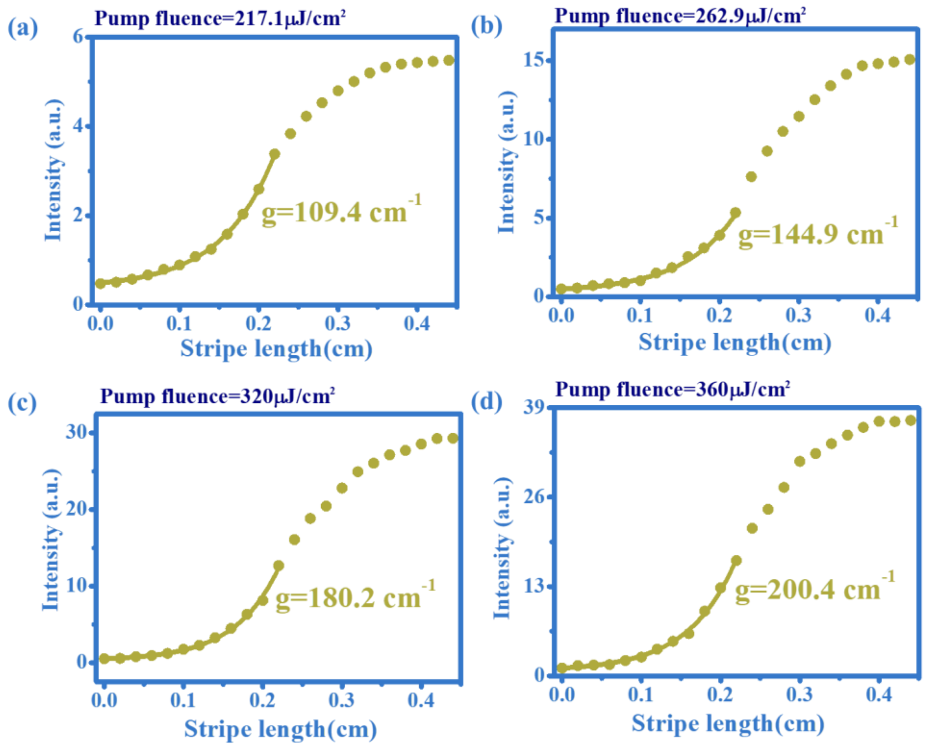

Figure S15 ASE peak intensity as a function of stripe length for sample S-II under excitation pump fluence of (a)-(d)

Flexible ASE measurement of S-III/PET

In this section, we investigated the flexible ASE and PL behaviors of S-III/PET, which was fabricated by embedding $FAPbI_3$ NCs between two PMMA layers. Figure S16(a) shows the evolution of ASE spectra during the bending process, clearly revealing variations in emission peak wavelength and intensity under different curvatures. Figure S16(b) presents the dependence of the ASE peak wavelength (blue line) and intensity (red line) on curvature. It shows that the ASE peak evolution of S-III under bending exhibits a trend similar to that of S-II. As the curvature increases from -0.91 cm^{-1} (concave) to +0.91 cm^{-1} (convex), the ASE peak wavelength redshifts from 801.1 nm to 807.5 nm, accompanied by a maximum intensity enhancement of 3.35 times.

Notably, both the intensity enhancement and the wavelength shift are more pronounced in S-III/PET than in S-II/PET. This can be attributed to the symmetric double-layer PMMA structure, which significantly enhances the waveguiding effect and thereby influences the ASE behavior under bending. Furthermore, the bending-induced PL spectral variation for S-III/PET is shown in Figure S16(c). The corresponding intensity variation and wavelength shift with curvature in Figure S16(d) exhibit a trend similar to that in Figure S16(b). This implies that mechanical bending enhances light scattering, which in turn contributes to the increased ASE and PL intensities.

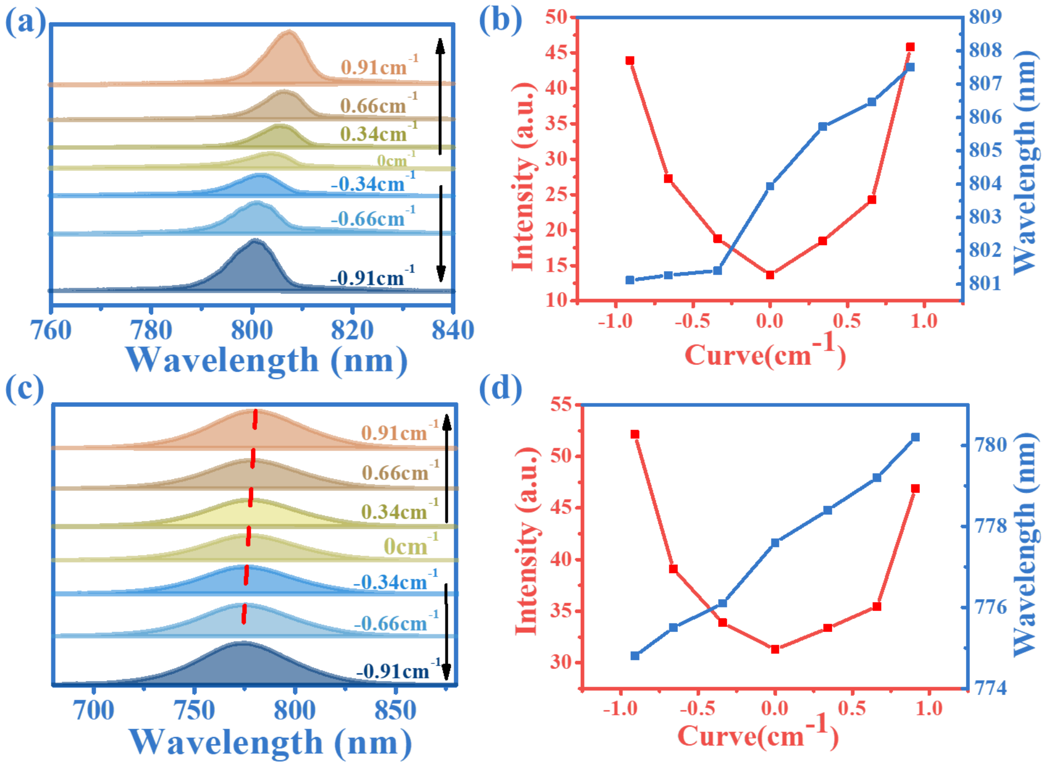

Figure S16. Flexible ASE and PL of bent $FAPbI_3$ NC film S-II/PET and S-III/PET under mechanical stress. (a) Evolution of ASE spectra as the sample is bent from the concave to the convex state, (b) corresponding ASE peak intensity and wavelength as functions of curvature, (c) evolution of PL spectra as the sample is bent from the concave to the convex state, and (d) corresponding PL peak intensity and wavelength as functions of curvature.

Reference:

(1) Huang, Z.; Qin, H.; Wen, J.; Jiang, L.; Hu, G.; Li, M.; Chen, J.; Liu, F.; Tang, T. Observation of abnormal photoluminescence upon structural phase competence and transition-induced disorder of stable α-FAPbI3. *Optical Materials Express* **2022**, *13* (1). DOI: 10.1364/ome.477818.

(2) Papagiorgis, P.; Manoli, A.; Protesescu, L.; Achilleos, C.; Violaris, M.; Nicolaides, K.; Trypiniotis, T.; Bodnarchuk, M. I.; Kovalenko, M. V.; Othonos, A.; et al. Efficient Optical Amplification in the Nanosecond Regime from Formamidinium Lead Iodide Nanocrystals. *ACS Photonics* **2018**, *5* (3), 907–917. DOI: 10.1021/acsphotonics.7b01159.

(3) Fang, H. H.; Wang, F.; Adjokatse, S.; Zhao, N.; Even, J.; Antonietta Loi, M. Photoexcitation dynamics in solution-processed formamidinium lead iodide perovskite thin films for solar cell applications. *Light Sci Appl* **2016**, *5* (4), e16056. DOI: 10.1038/lsa.2016.56.

(4) Wright, A. D.; Verdi, C.; Milot, R. L.; Eperon, G. E.; Perez-Osorio, M. A.; Snaith, H. J.; Giustino, F.; Johnston, M. B.; Herz, L. M. Electron-phonon coupling in hybrid lead halide perovskites. *Nat Commun* **2016**, *7*. DOI: 10.1038/ncomms11755.

(5) Fang, H. H.; Protesescu, L.; Balazs, D. M.; Adjokatse, S.; Kovalenko, M. V.; Loi, M. A. Exciton Recombination in Formamidinium Lead Triiodide: Nanocrystals versus Thin Films. *Small* **2017**, *13* (32). DOI: 10.1002/smll.201700673.
